# Exploring the neuroprotective potential of ligustrazine: a preclinical meta-analysis and machine learning perspective on cerebral ischemia-reperfusion injury

**DOI:** 10.3389/fnins.2026.1754510

**Published:** 2026-05-11

**Authors:** Lang Tu, Yimiao Luo, Xinyi Xu, Huiling Xiong, Zilong Zhang, Xiaoyuan Zhou

**Affiliations:** 1 West China School of Public Health / West China Fourth Hospital, Sichuan University, Chengdu, China; 2 School of Chinese Medicine, The University of Hong Kong, Hong Kong, Hong Kong SAR, China; 3 School of Public Health, Chengdu University of Traditional Chinese Medicine, Chengdu, China; 4 Acupuncture and Tuina School, Chengdu University of Traditional Chinese Medicine, Chengdu, China

**Keywords:** cerebral ischemia-reperfusion injury, ligustrazine, machine learning, meta-analysis, preclinical evidence

## Abstract

**Objective:**

This study aimed to assess the efficacy of ligustrazine in treating cerebral ischemia-reperfusion (I/R) injury and construct a preclinical evidence framework by meta-analysis and machine learning.

**Methods:**

A systematic search was conducted for preclinical studies published in PubMed, Embase, Web of Science, and the Cochrane Library up to June 25, 2024. The inclusion criteria encompassed preclinical animal studies pertinent to the topic. Data extraction was performed independently by two individuals, Stata 17.0 software was used for quantitative analysis, R (version 4.3.3) and Python (version 3.11.4) were used for machine learning with neurological function score as the dependent variable.

**Results:**

A total of 23 articles were included, involving 381 animals in the meta-analysis and 321 animals in the machine learning component. Ligustrazine significantly improved neurofunctional scores (NFS) [Longa criteria, *SMD* = −1.59, *95%CI* (−2.16, −1.01), *P* < 0.001; mNSS criteria, *SMD* = −1.67, *95%CI* (−2.36, −0.97), *P* < 0.001], cerebral infarct volume (%) [*SMD* = −2.56, *95%CI* (−3.03, −2.09), *P* < 0.001], and BBB [*SMD* = −3.06, *95%CI* (−4.53, −1.59), *P* < 0.001]. Furthermore, machine learning analyses, with NFS as the dependent variable, identified the time of first dose, duration, and dose as key determinants of neurofunctional improvement with ligustrazine. Notably, model interpretation suggested that greater improvements were more likely to occur when the initial administration of ligustrazine occurred within 24 h prior to (or 2.21 h post) the ischemic event, at a dosage of 23.53–34.69 mg/kg/day (or 45.71 to 75.65 mg/kg/day), and with an administration duration exceeding 71.43 h.

**Conclusion:**

The combination of meta-analysis and machine learning in this study not only confirms that ligustrazine is effective in reducing cerebral I/R injury, but also provides a framework for elucidating the preclinical intervention variables, thus offering novel insights for optimizing preclinical strategies of ligustrazine in cerebral I/R injury.

## 1 Introduction

Stroke, particularly ischemic stroke, is a prevalent global health issue contributing significantly to mortality and disability, which has become increasingly prominent with the aging population (Feigin et al., 2017; Katan and Luft, 2018). Despite advances in vascular recanalization strategies, cerebral damage following reperfusion remains a major challenge. Research has shown that ischemic stroke leads to neuronal cell damage via mitochondrial dysfunction, oxidative stress, apoptosis, cytokine production, and inflammation. Revascularization further exacerbates oxidative stress, promoting the release of pro-inflammatory cytokines that initiate a cascade of reactions, leading to blood-brain barrier (BBB) breakdown, brain edema, and hemorrhagic transformation (Jurcau and Simion, 2021; Tuo et al., 2022; Huang et al., 2023). Previous studies have explored the preventive and therapeutic effects of promising monomers such as resveratrol in cerebral I/R injury (Yang et al., 2022).

Small molecular monomers derived from natural products, with their characteristics of multitarget comprehensive treatment and holistic regulation, have exhibited encouraging results in both preventing and treating a wide range of diseases (Hu et al., 2023). These unique characteristics make natural monomer drugs particularly promising for certain diseases, including cerebral I/R injury (Tabrizi et al., 2019). Among these monomers, tetramethylpyrazine (TMP, PubChem CID: 14296) is particularly concerned by the research community for its improvement on cerebral I/R injury, which plays a comprehensive role through a variety of mechanisms such as anti-inflammatory, anti-apoptosis, and anti-oxidative stress. For this monomer, which extracted from *Ligusticum wallichii Franchat*, a systematic review concluded the potential mechanisms of anti-cerebral I/R injury in 2015(Gao et al., 2015). However, the clinical importance of cerebral I/R injury and the underlying medicinal value of TMP prompted further exploration, and to date, there have been at least ten relevant preclinical studies to explore this aspect of TMP more richly (for example, more comprehensive studies of TMP on BBB permeability and cell synapses were carried out) since the above article was published (Tan et al., 2015; Yu B. et al., 2016; Liao et al., 2018; Ding et al., 2019; Cao et al., 2020; Jin et al., 2021; Lin et al., 2021; Teng et al., 2021; Li et al., 2022; Mu et al., 2023; Chen et al., 2024; Shu et al., 2024). These studies have further enriched the related mechanisms and pathways of TMP in the treatment of cerebral ischemia-reperfusion injury.

Despite these promising results, significant heterogeneity exists across studies, making it essential to derive more consistent conclusions through a meta-analysis (Siddaway et al., 2019). Furthermore, although clinical meta-analyses of TMP in cerebral infarction exist (Yu T. et al., 2016), a preclinical meta-analysis rigorously summarizing the mechanisms of TMP is notably absent. In the article by Wang et al. (2024), the therapeutic effect of ligustrazine on ischemic stroke was meta-analyzed. However, our article focuses on I/R injury in ischemic stroke to elucidate the mechanisms of cerebral I/R injury more clearly. Additionally, from a broader perspective, traditional meta-analyses often face challenges in integrating cutting-edge technologies. For example, meta-analysis often stop short at drawing static conclusions from aggregated data, without leveraging methods that allow for further quantitative predictions or personalized insights. In contrast, our study addresses these gaps by incorporating machine learning into the meta-analysis process. This approach not only enhances the precision of the dose-efficacy analysis but also allows for a dynamic understanding of the factors influencing therapeutic outcomes. By combining rigorous methodological standards with innovative techniques, we aim to provide a more structured and forward-thinking framework for future research in this field.

In recent years, several systematic reviews and meta-analyses have assessed the efficacy of ligustrazine in the treatment of ischemic stroke. For example, Wang et al. (2024) conducted a preclinical meta-analysis of ligustrazine in ischemic stroke models and highlighted its anti-inflammatory, antioxidant, and anti-apoptotic effects. Similarly, Shao et al. (2021) and Yu T. et al. (2016) evaluated the clinical efficacy of ligustrazine injection as an adjuvant therapy for acute cerebral infarction and reported improvements in neurological function and hemorheological parameters. However, these studies mainly focus on the overall outcome of ischemic stroke or acute clinical efficacy, but did not specifically addressed I/R injury, a pathological process with unique mechanistic significance. Furthermore, these studies have largely relied on traditional meta-analysis methods, which have limited ability to model complex multifactorial treatment responses or to predict individualized therapeutic time windows. In contrast, this study extends previous work in several aspects. First, it focuses specifically on preclinical models of cerebral I/R injury to better elucidate the underlying pathological mechanisms. Second, it integrates machine learning algorithms with meta-analysis data to construct models between intervention parameters (e.g., dose, timing, and duration) and neurological outcomes. Third, it applies data-driven approaches to identify potential optimal dose ranges and therapeutic time thresholds, thereby providing a more quantitative reference framework for future translational research.

Based on the above considerations, this study proposes a novel methodological framework that integrates traditional evidence synthesis with machine learning techniques. This approach aims to enhance the interpretability and translational value of preclinical evidence and to provide a more objective and systematic reference for future studies.

## 2 Materials and methods

### 2.1 Meta analysis and systematic review

The meta-analysis adhered to the PRISMA guidelines, ensuring a systematic and rigorous approach.

#### 2.1.1 Protocol and registration

The protocol was registered and the following procedures were performed followed from the registered INPLASY protocol. The transparency of the conduct was ensured, and the bias risk of the articles was minimized. The INPLASY registration number was 202360002 (doi: 10.37766/inplasy2023.6.0002).

#### 2.1.2 Data and retrieval formula

Four databases (Embase, the Cochrane Library, PubMed, Web of Science) were retrieved from inception to June 25, 2024. Search terms included “ligustrazine” or “chuanxiongzine”, “cerebral I/R injury”, etc., and the detailed retrieval formula was presented in Supplementary Table 1.

Retrieved records were screened independently by two researchers according to the eligibility criteria. Any disputes were firstly discussed between the two of them, and if the disputes could not be resolved, these disputes were referred to the corresponding author for adjudication.

#### 2.1.3 Eligibility criteria

According to the PICOS principles, the inclusion and exclusion criteria for this meta-analysis were as follows:

For the inclusion criteria: (1) Population (P): study was a rodent experiment (mice or rats) with cerebral I/R injury. (2) Intervention (I): the experimental group was treated with TMP. (3) Comparison (C): equal doses of saline or vehicle were administered to animals in model group. (4) Outcome (O): primary and secondary outcome measures were used to evaluate the therapeutic role of TMP on this disease, etc. (5) Study designs (S): preclinical studies.

For the exclusion criteria: (1) Population (P): article was not a rodent experiment and/or a non-cerebral I/R injury model. (2) Intervention (I): experimental group did not utilize ligustrazine alone and/or a monomer, etc. (3) Comparison (C): not a blank control group. (4) Outcome (O): the articles had no relevant data or could not be extracted. (5) Study designs (S): not original articles.

#### 2.1.4 Data extraction

Custom data sheets were used to extract data independently by two investigators (TU L and Luo YM). The contents of the customized data sheet were as follows: (1) basic features of included literature, including publication year and author. (2) characteristics of the animals used in the experiment, including species, weight, sex, and number of rodents. (3) time of stroke model establishment and reperfusion. (4) *P*-value of ligustrazine in the treatment of stroke in each dose group and model group. (5) main outcomes in each study.

Regarding the data for indicators, all of them were extracted from two or more studies. Only the maximum dose and duration of treatment were considered when faced with multiple different doses and/or durations. In cases where data were not directly available, attempts were made to contact the authors to obtain relevant data, and the ruler tool was used in cases in which contact with the authors was unsuccessful. Mean, standard deviation (*SD*), or standard error (*SEM*) of the indexes were extracted. If the article was expressed by the standard error, the standard error would be converted to standard deviation during data analysis by the following formula: *SD* = $SEM^{*}\sqrt{n}$ (Lee et al., 2015).

#### 2.1.5 Quality assessment of included articles

CAMARADES 10-point scale was an international standard published in 2004, which was used to assess the articles' quality (Macleod et al., 2004). Meanwhile, some options on the scale have been modified to make it more applicable to this study (D: blinded induction of ischemia; F: avoid the use of anesthetics with significant intrinsic neurotoxicity; G: cerebral I/R injury model). Two researchers (Xu XY and Xiong HL) independently applied the scale to assess methodological quality of articles and associated bias. In case of disagreement, the corresponding author adjudicated to reach consensus.

#### 2.1.6 Statistical analyses

Quantification of the included parameters was performed using 95% confidence intervals (*95%CI*) and standardized mean differences (*SMD*). For *SMD* values, when the value was greater than (less than) 0, it indicated that ligustrazine could promote (inhibit) the change of this index. Furthermore, *SMD* values were statistically significant when their *95%CI* did not include zero. The results of histological examination were further observed to determine whether it had clinical significance.

To account for possible heterogeneity in the results, we determined and quantified heterogeneity using the chi-square test and *I*^2^. If *I*^2^ < 50% (or *I*^2^> 50%), the fixed-effect model (or random-effect model) was used to combine the data because of small (or high) heterogeneity. On this basis, subgroup analysis and sensitivity analysis were applied to appropriate indicators as far as possible to ensure the stability of the results.

Publication bias was assessed by Egger's test for the main outcome measures. When |t| < 0.05 (or |t|> 0.05), indicated a significant publication bias (or without). when |t| < 0.05, bias could be further validated by trim and fill method.

Stata 17.0 was used to perform quantitative synthesis and statistical analysis. Moreover, Origin 2022 was used to draw the three-dimensional graph of the data structure.

#### 2.1.7 Summary of potential mechanisms

Potential mechanisms of ligustrazine therapeutic effects were summarized based on the available articles, including the regulation of cerebral blood flow, anti-oxidative stress, anti-inflammatory, anti-apoptosis, and other mechanisms.

A comprehensive and reliable summary was performed by investigating the efficacy and potential mechanisms of TMP, which improving cerebral I/R injury.

### 2.2 Machine learning

Considering that conventional meta-regression primarily models simple linear relationships and may have limited ability to capture complex interactions among multiple experimental parameters, a machine-learning approach was employed in this study to explore potential nonlinear associations within the dataset. Machine learning provides a flexible framework for modeling multidimensional relationships and identifying key predictors of treatment response. In addition, SHapley Additive explanation (SHAP) was applied to interpret the model and quantify the contribution of each variable to the predicted outcomes, thereby enhancing the interpretability of the machine-learning results.

#### 2.2.1 The research object and variable selection

The same inclusion and exclusion criteria outlined in Section 2.1. Because sample sizes vary across studies, this can introduce potential biases that affect machine-learning results, for data consistency, relevant sample data were averaged for each document (Guo et al., 2024). To evaluate the therapeutic effect of ligustrazine, intervention doses, durations, time of first administration, ischemia time and reperfusion time were collected as independent variables. Meanwhile, NFS improvement rate was selected as the dependent variable to better evaluate the therapeutic effect of ligustrazine on cerebral I/R injury, which was the most included indicator in all studies. NFS improvement rate = [(|mean of control group – mean of experimental group|)/ mean of control group] ^*^100%.

#### 2.2.2 Processing of data

The data set consists of 321 samples exhibiting significant variability. To enhance the data's structural robustness, normalization was applied, standardizing sample means to 0 and standard deviations to 1 (De Livera et al., 2015). This approach simplifies the distribution of the data, boosts the efficacy of model training with these samples, reduces the training complexity, and improves the model's interpretability.

#### 2.2.3 Development and assessment of predictive models

In order to avoid data leakage and overfitting problems, we adopted a strict data partitioning strategy: all valid data were randomly divided into training set and test set at a ratio of 7.5:2.5 to ensure that the test set was completely independent during the training process. In order to ensure the generalization ability of the model, five-fold cross validation was adopted. Different machine learning models exhibit distinct inductive biases, which can significantly influence the training process of a dataset. Given the dataset's limited size and the continuous nature of the dependent variable, models such as Multilayer Perceptron (MLP), Simple Hidden Layer Neural Network (SHLNN), and Extreme Gradient Boosting (XGBoost)—known for their efficacy with analogous datasets—were selected. Additionally, a stacked ensemble learning approach was implemented to precisely forecast the NFS improvement rate. The data were normalized to enhance the dataset's integrity prior to analysis. Posterior to the development of individual models, a grid search technique was applied to calibrate the hyperparameters, subsequently assessing their performance via cross-validation. To enhance the ensemble's precision, Lasso (Least Absolute Shrinkage and Selection Operator) regression was deployed to refine the meta-model's features. The ensemble model was subsequently applied to both the training and test datasets to generate predictions and conduct evaluations. A comprehensive assessment of the Stacking Ensemble Model's performance was conducted using metrics including Root Mean Squared Error (RMSE), R-squared (R^2^), and Mean Absolute Error (MAE).

#### 2.2.4 Model interpretation

Since machine learning is uncertain and can be difficult to interpret, we rely on the game theory-based SHAP method, proposed by Lundberg to accurately interpret the model outputs (Lee et al., 2017). The SHAP approach ranks the importance of features in the input data based on their SHAP values: higher SHAP values indicate a correspondingly greater positive influence of that feature on the machine learning model, whereas lower SHAP values suggest a smaller or even negative impact.

The software utilized in this research comprises R (version 4.3.3) and Python (version 3.11.4).

## 3 Results

### 3.1 Literature screening

**Figure 1:**
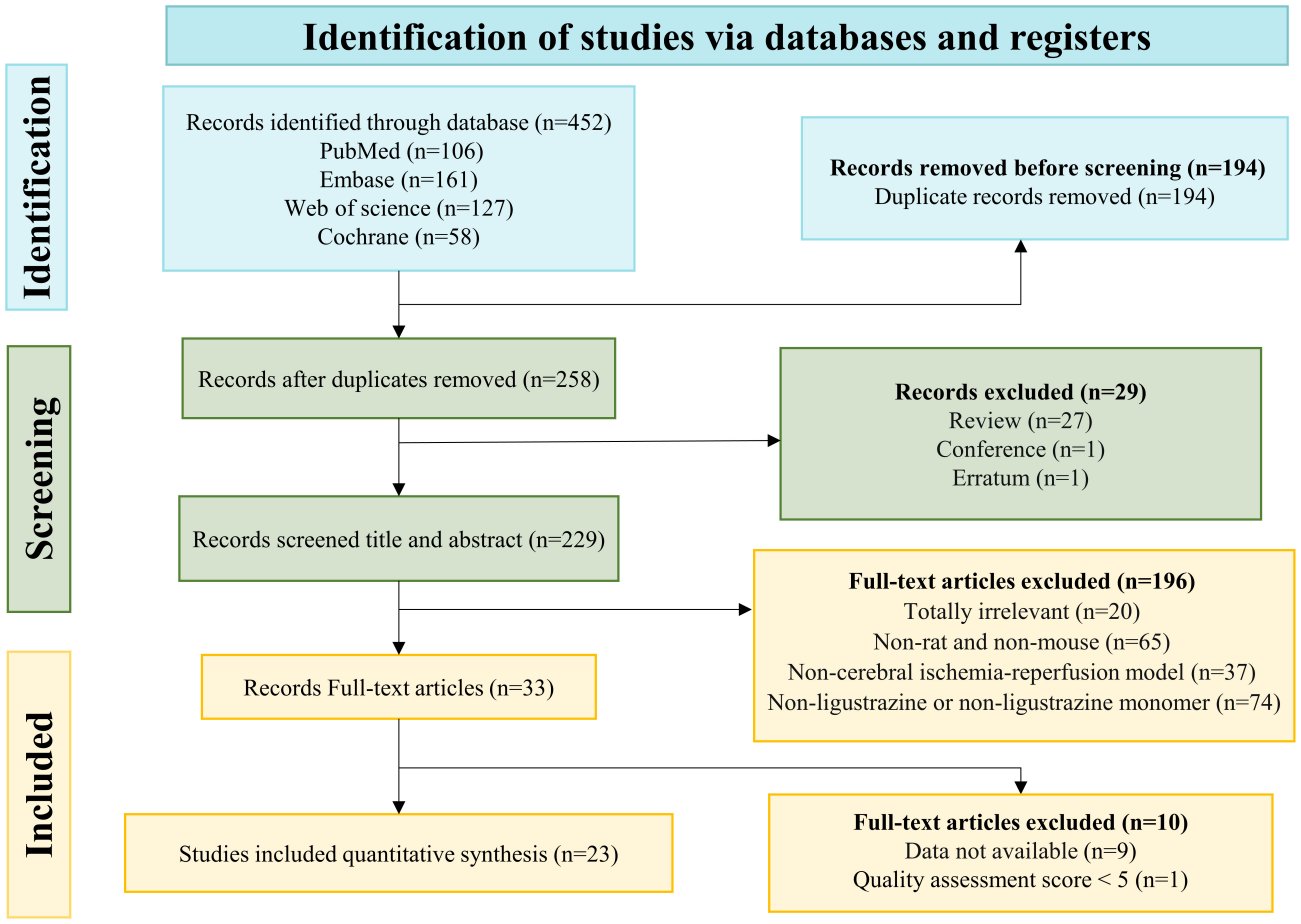
Detailed process of included articles.

A complete search formula was used, and 452 potentially relevant records were retrieved. Firstly, 223 records were excluded before reading titles and abstracts, included duplicate records, reviews, and conference abstracts, etc. Subsequently, we reviewed the titles and abstracts of the remaining 229 records carefully, 196 records were not considered because they contained any one of the exclusion criteria: (1) non-rat/mouse experiments (*n* = 65); (2) non-cerebral ischemia-reperfusion injury (*n* = 37); (3) non-ligustrazine monomer (*n* = 74); (4) totally irrelevant (*n* = 20). The remaining 33 articles were downloaded, and meticulous review was achieved by reading the full text. Although 10 articles met the inclusion criteria, they were excluded due to the lack of available data and low-quality. Ultimately, 23 articles eligible for this meta-analysis were left for quantitative analysis (Liao et al., 2004, 2018; Hsiao et al., 2006; Chang et al., 2007; Qi et al., 2007a,b; Jia et al., 2009; Yang et al., 2009; Zhu et al., 2009; Xiao et al., 2010; Han et al., 2014; Lin et al., 2015, 2021; Tan et al., 2015; Yu B. et al., 2016; Ding et al., 2019; Cao et al., 2020; Jin et al., 2021; Teng et al., 2021; Li et al., 2022; Mu et al., 2023; Chen et al., 2024; Shu et al., 2024) (Figure 1).

### 3.2 Characteristics of included articles

**Figure 2:**
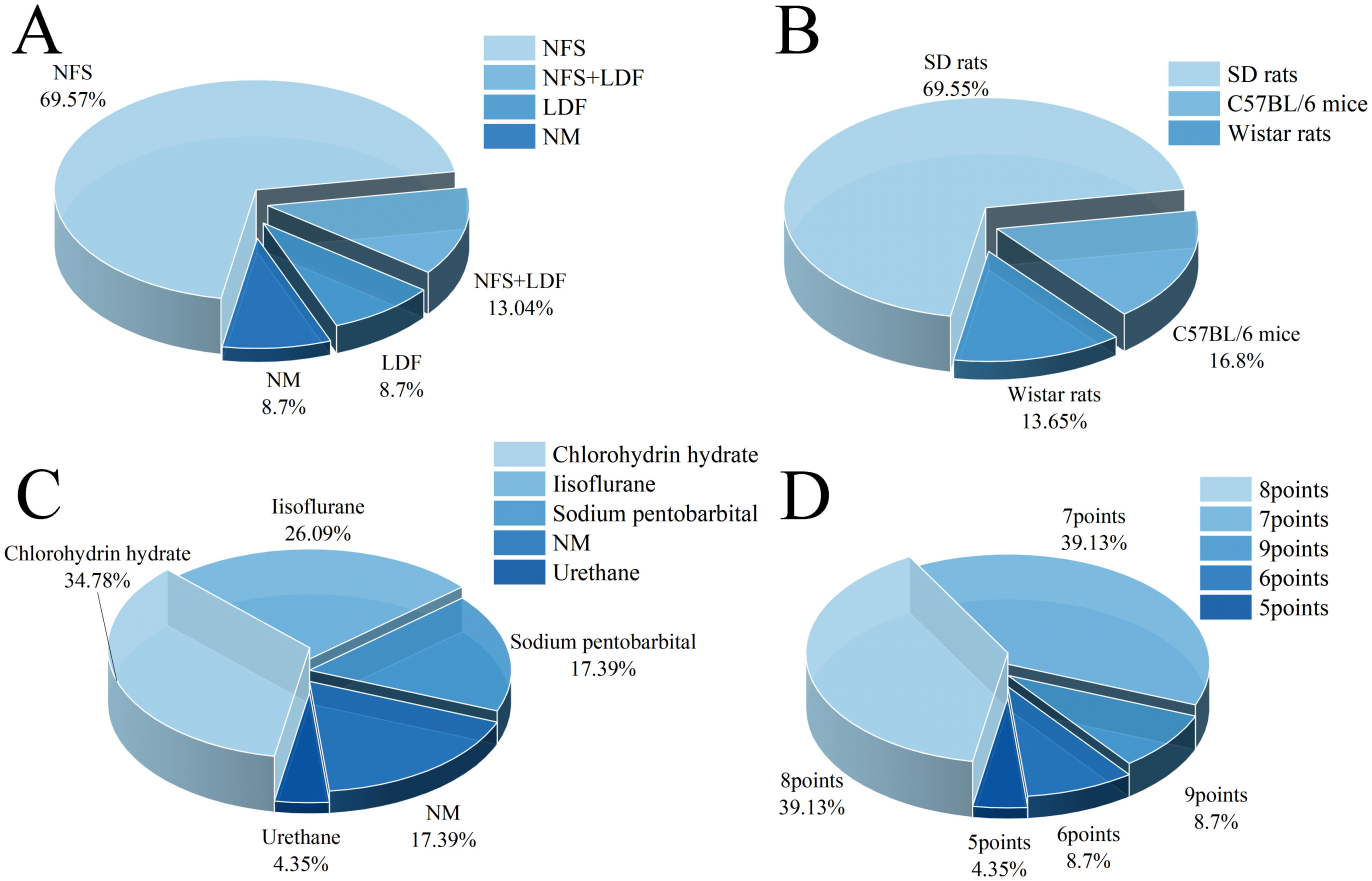
Relevant characteristics of included articles. **(A)** Confirmation of successful modeling methods; **(B)** Species distribution of rodents; **(C)** Anesthetic types; **(D)** Article quality score.

In the 23 remaining articles, the longest time of cerebral ischemia was 2 h, and the minimum time of reperfusion was 6 h. For the modeling of animals, neurological function scores were used to evaluate the status of modeling in 16 articles, and animals that did not reach the corresponding scores were excluded. In addition, laser Doppler flowmetry (LDF) was used to determine the success of model in 8.70% (2/23) articles, and 13.04% (3/23) used neurological function scores and LDF to determine the success of cerebral I/R injury in the animals (Figure 2A).

This study included a number of 381 animals, 185 of them were in experimental group, another 196 were in model group. All the included articles were male rodents except 1 article did not mention the gender, and 80.58% (307/381) of the rodents were rats, and most of the rats were Sprague-Dawley rats (Figure 2B). Based on the inclusion/exclusion criteria, the experimental groups only received with ligustrazine, the highest dose was 80 mg/kg, the lowest dose was 10 mg/kg, and same amount of saline or vehicle were administered to model group. Most of the included studies used intraperitoneal injection of ligustrazine, and a few used oral administration or tail vein injection. In terms of anesthesia, chlorohydrin hydrate was used in 8 articles, 26.09% (6/23) was used isoflurane mixed with oxygen and carbon dioxide, sodium pentobarbital was used in 4 articles (Figure 2C).

For the primary outcome indexes, pathological examination was reported in 82.61% (19/23) of the articles. NFS was reported in 60.87% (14/23) articles, followed by 11 (47.83%) articles on cerebral infarction volume percentage, 17.39% (4/23) articles on cerebral edema, and 13.04% (3/23) articles on blood-brain barrier permeability (Table 1).

**Table 1:** Key characteristics of the 23 included articles.

References	Anesthetic (dose/mode of administration)	Sex/species (*n =* TMP/Con)	Weight	Model	Administration dose/mode/time of TMP	Outcome indexes
Liao et al. (2004)	Chloral hydrate (400 mg/kg, IP)	Male Sprague–Dawley rats (4/7)	300–350g	Ischemia for 1.5h, reperfusion for 3 d	40 mg/kg, IP, 1h before MCAO	1, 3, 4
Hsiao et al. (2006)	3% isoflurane (inhalation)	Male Wistar rats (8/10)	250–300g	Ischemia for 1h, reperfusion for 24h	20 mg/kg, IP, 20min before MCAO	1
Chang et al. (2007)	3% isoflurane (inhalation)	Male Wistar rats (8/14)	250–300g	Ischemia for 1h, reperfusion for 24h	20 mg/kg, IP, 20min before MCAO	1, 5, 6, 7
Qi et al. (2007a)	NM	Male Sprague–Dawley rats (6/6)	260–300g	Ischemia for 2h, NM reperfusion time	40 mg/kg/d, IP, 20min after MCAO	8, 9
Qi et al. (2007b)	NM	Male Sprague–Dawley rats (6/6)	260–300g	Ischemia for 2h, NM reperfusion time	40 mg/kg/d, IP, 20min after MCAO	9, 10, 20, 21
Jia et al. (2009)	Chloral hydrate (400 mg/kg, IP)	Male Sprague–Dawley rats (8/8)	300–350g	Ischemia for 2h, reperfusion for 72h	20 mg/kg/d, IP, 1h after reperfusion	2, 8, 22, 23, 24, 25
Yang et al. (2009)	NM	Male Sprague–Dawley rats (6/6)	280 ± 20g	Ischemia for 2h, reperfusion for 24h	40 mg/kg, IP, 12h before MACO	1, 8, 11
Zhu et al. (2009)	2% isoflurane (inhalation)	Male Sprague–Dawley rats (8/8)	300–350g	Ischemia for 2h, reperfusion for 72h	20 mg/kg/d, IP, 1h after MACO	2, 8, 22, 23, 24, 25
Xiao et al. (2010)	Sodium pentobarbital (NM, IP)	Male Sprague–Dawley rats (6/6)	180–220g	Ischemia for 2h, NM reperfusion time	40 mg/kg/d, IP, NM	2, 10
Han et al. (2014)	Urethane (1250 mg/kg, IP)	Male Sprague–Dawley rats (10/10)	290 ± 10g	Ischemia for 1h, reperfusion for 2h	50 mg/kg, NM, NM	2, 8
Lin et al. (2015)	10% chloral hydrate (400 mg/kg, IP)	Male Sprague–Dawley rats (18/18)	200–250g	Ischemia for 2h, reperfusion for 3 d	20 mg/kg/d, IP, 2h after reperfusion	4, 8
Tan et al. (2015)	2% isoflurane (inhalation)	Male Sprague–Dawley rats (8/8)	250–270g	Ischemia for 1.5h, reperfusion for 22.5h	20 mg/kg, IP, 15min before MCAO	1, 3, 8, 13
Yu B. et al. (2016)	10% chloral hydrate (0.35 g/kg, IP)	Male Sprague–Dawley rats (10/10)	250–300g	Ischemia for 10min, NM reperfusion time	13.3 mg/kg/d, PO, NM	6, 11
Liao et al. (2018)	10% chloral hydrate (0.3 mg/kg, IP)	Male Sprague–Dawley rats (6/6)	235–265g	Ischemia for 2h, reperfusion for 22h	4mg/kg,IP, 40min before MACO	1
Ding et al. (2019)	10% chloral hydrate (350 mg/kg, IP)	Wistar rats (6/6)	NM	Ischemia for 2h, reperfusion for 24h	10 mg/kg, IP, 2h after MACO	8, 15, 16, 17
Cao et al. (2020)	10% chloral hydrate (inhalation)	Male Sprague–Dawley rats (10/10)	250–300g	Ischemia for 2h, reperfusion for 7 d	50 mg/kg/d, IP, 24h after MACO	8, 12, 18
Jin et al. (2021)	3% isoflurane (inhalation)	Male C57BL/6 mice (12/12)	22–25g	Ischemia for 2h, reperfusion for 24h	50 mg/kg, IP, 12h before MACO	3, 7, 13
Lin et al. (2015)	Sodium pentobarbital (50 mg/kg, IP)	Male Sprague–Dawley rats (10/10)	200–250g	Ischemia for 2h, NM reperfusion time	20 mg/kg/d, IP, 2h after MACO	8, 19
Teng et al. (2021)	Sodium pentobarbital (50 mg/kg, IP)	Male Sprague–Dawley rats (5/5)	260 ± 20g	Ischemia for 2h, NM reperfusion time	30 mg/kg/d, Tail vein, NM	1
Li et al. (2022)	10% chloral hydrate (0.5 mL/100g, IP)	Male C57BL/6 mice (5/5)	NM	Ischemia for 1h, reperfusion for 24h	20 mg/kg, IP, 15min after MACO	1, 8
Mu et al. (2023)	2% isoflurane (inhalation)	Male C57BL/6 mice (15/15)	20–25g	Ischemia for 1.5h, NM reperfusion time	20 mg/kg, Tail vein, 2 d after MACO	1, 2, 3, 8, 13
Shu et al. (2024)	NM	Male Sprague–Dawley rats (5/5)	230–250g	Ischemia for 2h, reperfusion for 24h	20 mg/kg, IP, 2h after MACO	1, 8
Chen et al. (2024)	2% pentobarbital sodium (50 mg/kg, IP)	Male Sprague–Dawley rats (5/5)	250–280 g	Ischemia for 2 h, reperfusion for 24 h	80 mg/kg, Gavage, 24 h before MACO	1, 8

1. Infarct volume (%); 2. infarct volume; 3. water content; 4. MAP-2; 5. HIF-α; 6. caspase-3; 7. TNF-α; 8. neurological function score; 9. DG Brdu-positive cells; 10. SVZ BrdU-positive cells; 11. MDA in the cerebral cortex; 12. The percentage of infarction size; 13. Blood-brain barrier permeability; 14. the contents of TNF-α in cortex; 15. NO levels in cells; 16. eNOS levels in cells; 17. p-eNOS levels in cells; 18. the weight of infarction size; 19. SYP expression; 20. nNOS expression at different time points in cortex; 21. nNOS expression at different time points in hippocampus; 22. Trx-1; 23. TrxR-1; 24. Trx-2; 25. TrxR-2.

DG, dentate gyrus; eNOS, endothelial nitric oxide synthase; HIF-α, Hypoxia-inducible factor α; IP, Intraperitoneal injection; MAP-2, anti-microtubule associated protein-2; MDA, malondialdehyde; NM, not mention; p-eNOS, phosphor endothelial nitric oxide; PO, Oral administration; SVZ, subventricular zone; TNF-α, tumor necrosis factor-α. synthase; SYP, synaptophysin; nNOS, neuro nitric oxide synthase; Tail vein, Tail vein injection; TrX-1, Thioredoxin-1; TrX-2, Thioredoxin-2; TrXR-1, thioredoxin reductase 1; TrXR-2, thioredoxin reductase 2; TMP, tetramethylpyrazine.

### 3.3 Literature quality

After the unified scale scoring of the included articles, it was found that the overall score of the included articles was 7.83, and the overall quality of the whole article was higher. Among them, the score of 2 articles was 9 points, more than 30 percent of the articles scored 8 points (39.13%, 9/23) or 7 points (39.13%, 9/23), and only 2 articles scored 6 points (Figure 2D).

**Figure 3:**
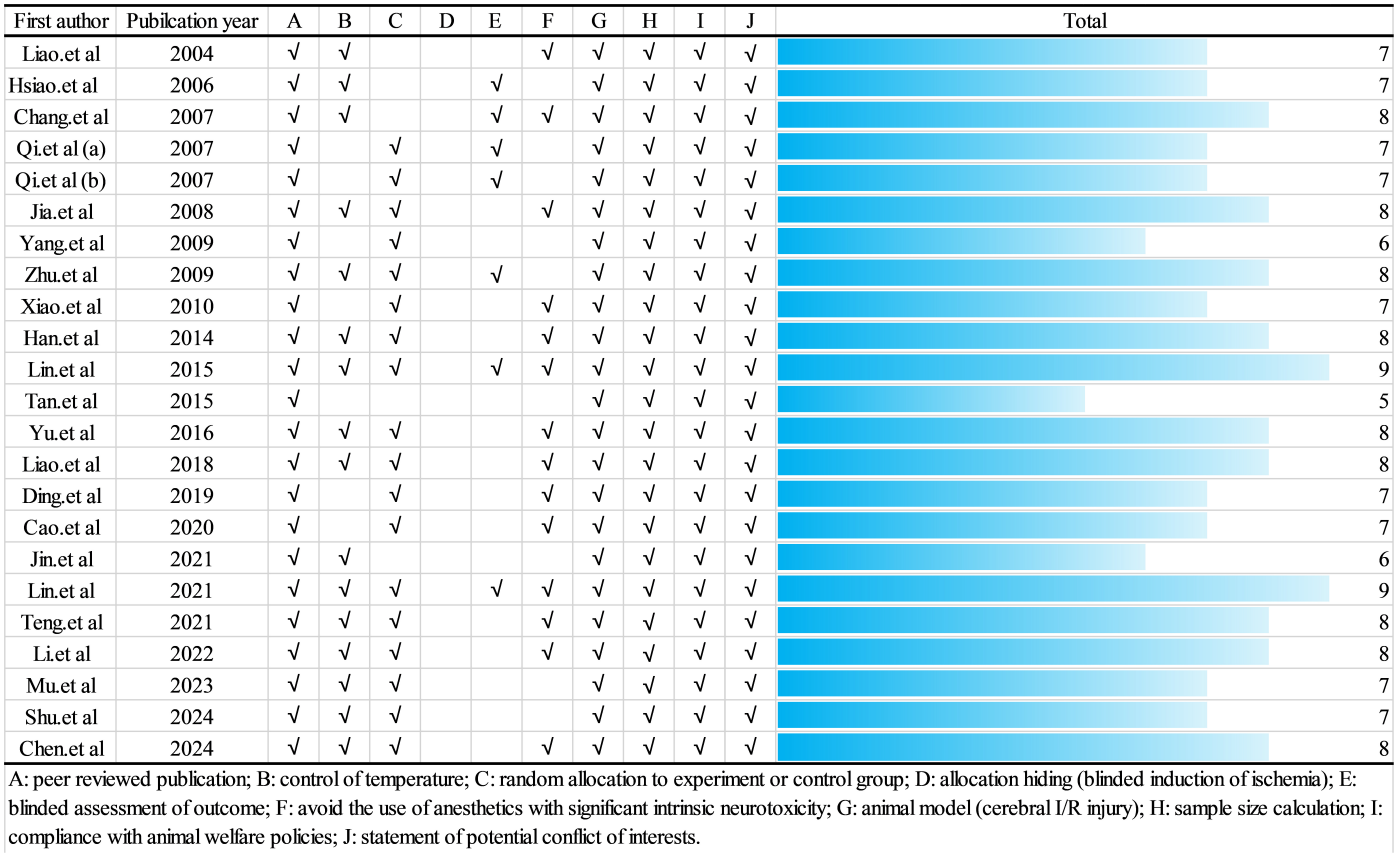
Risk of bias and quality assessment scores for each included article.

In general, all articles passed peer review and sample size calculation, and all the animal models were cerebral I/R injury in eligible articles. To avoid the information bias caused by the subjective factors of the experimenter, 7 articles evaluated the results in a blinded manner. Although 19 articles used random assignment to further reduce selection bias of animals, no specific randomization method was proposed. Seven articles did not mention whether temperature was controlled in laboratory (Figure 3).

### 3.4 Primary outcomes

#### 3.4.1 Pathological examination

Pathological examination could visually understand the therapeutic effect of ligustrazine on cerebral I/R injury animals. Among these studies, hematoxylin-eosin (H&E) staining (*n* = 4) observed nuclear atrophy, cell edema, inflammatory infiltration, and neuronal necrosis in animals with cerebral I/R injury. The number of surviving neurons increased. Changes in cerebral infarct volume were evaluated by triphenyl tetrazolium chloride (TTC) staining. In contrast to the sham group, this index in model group was significantly increased, while the cerebral infarct volume of ligustrazine group was effectively reduced. Eight articles used immunohistochemical staining, found that ligustrazine led to a reduction in the number of astrocytes after injury, while it promoted the generation of neurons and upregulated the expression of synaptophysin, which increased the number and function of synapses. The results of transmission electron microscopy in 2 articles showed that ligustrazine could effectively remodel synaptic ultrastructure, including improving synaptic interface flattening and thinning postsynaptic density of nerve cells after injury.

#### 3.4.2 Neurological function scores

Neurological function was scored by Longa or mNSS criteria in all included studies.

**Figure 4:**
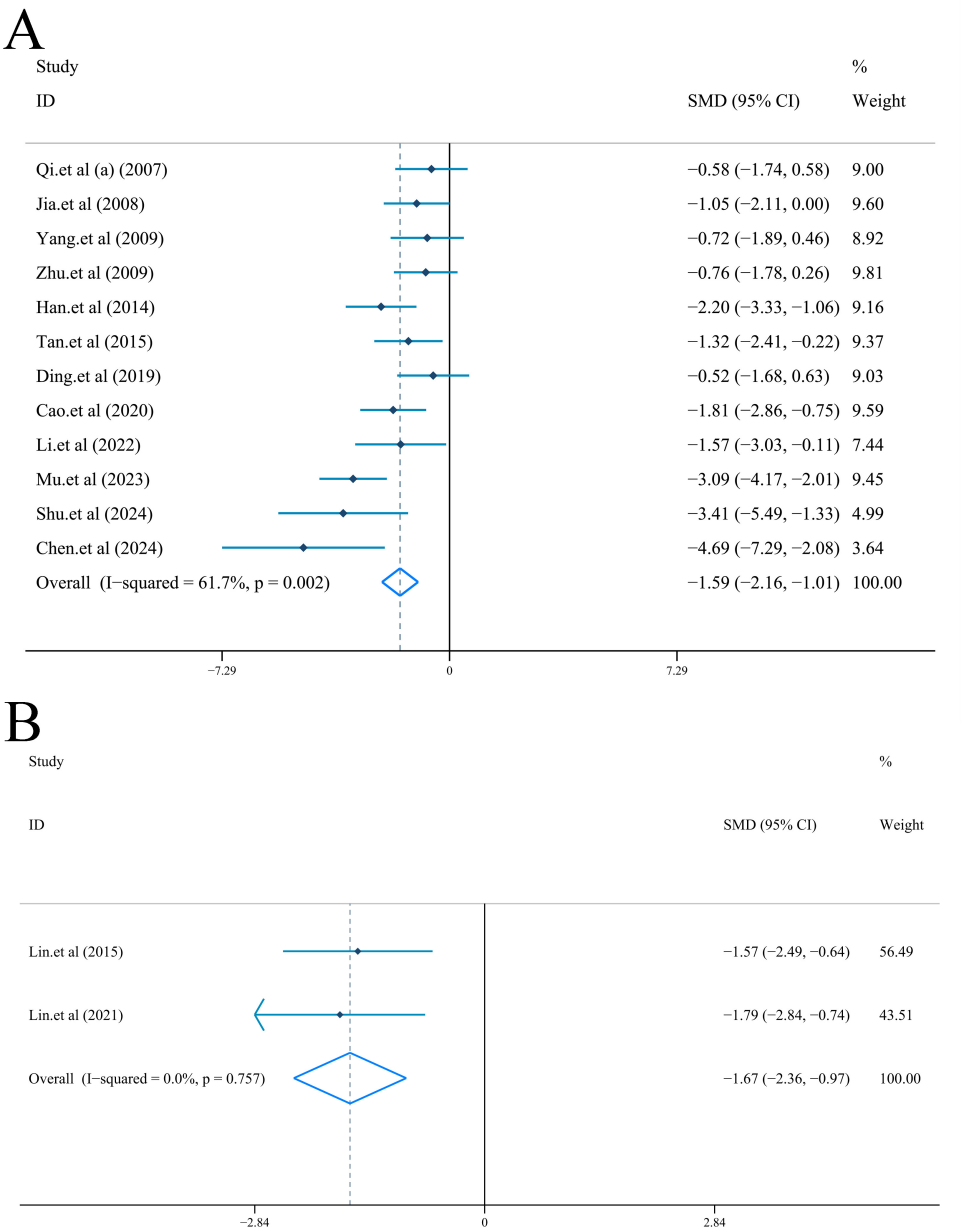
Forest plot (effect size and 95% CI) summarizing the effect of Ligustrazine on neurological function scores. **(A)** neurological function scores based on Longa criteria; **(B)** neurological function scores based on mNSS criteria.

Neurological function scored by Longa criteria: Random-effect model (*I*^2^ = 61.7%, *P* = 0.002) was used to combine the data from 12 articles (Qi et al., 2007b; Jia et al., 2009; Yang et al., 2009; Zhu et al., 2009; Han et al., 2014; Tan et al., 2015; Ding et al., 2019; Cao et al., 2020; Li et al., 2022; Mu et al., 2023; Chen et al., 2024; Shu et al., 2024). Compared with model group, results of NFS was significantly reduced [*n* = 184, *SMD* = −1.59, *95%CI* (−2.16, −1.01), *P* < 0.001] (Figure 4A). Furthermore, the species of subgroup analysis showed that ligustrazine was effective in reducing NFS in both rats [*n* = 144, *SMD* = −1.39, *95%CI* (−1.96, −0.82), *P* < 0.001; *I*^2^ = 53.0%, *P* = 0.024] and mice [*n* = 40, *SMD* = −2.41, *95%CI* (−3.90, −0.93), *P* = 0.001; *I*^2^ = 63.1%, *P* = 0.100] (Supplementary Figure 1A). Meanwhile, no significant publication bias was revealed by the test (|t| = 1.92>0.05, Supplementary Figure 2A). Sensitivity analysis showed thar after removing any one of the 12 studies, the combined results of the remaining 11 studies were still consistent with the original pooled results (Supplementary Figure 2B).

Neurological function scored by mNSS criteria: Two studies reported neurological deficits with mNSS criteria (Lin et al., 2015, 2021). Meta-analysis based on fixed-effect model [*I*^2^ = 0.0%, *P* = 0.757] indicated that ligustrazine could improve the degree of neurological injury [*n* = 44, *SMD* = −1.67, *95%CI* (−2.36, −0.97), *P* < 0.001] (Figure 4B).

#### 3.4.3 Infarct volume (%) and infarct volume

**Figure 5:**
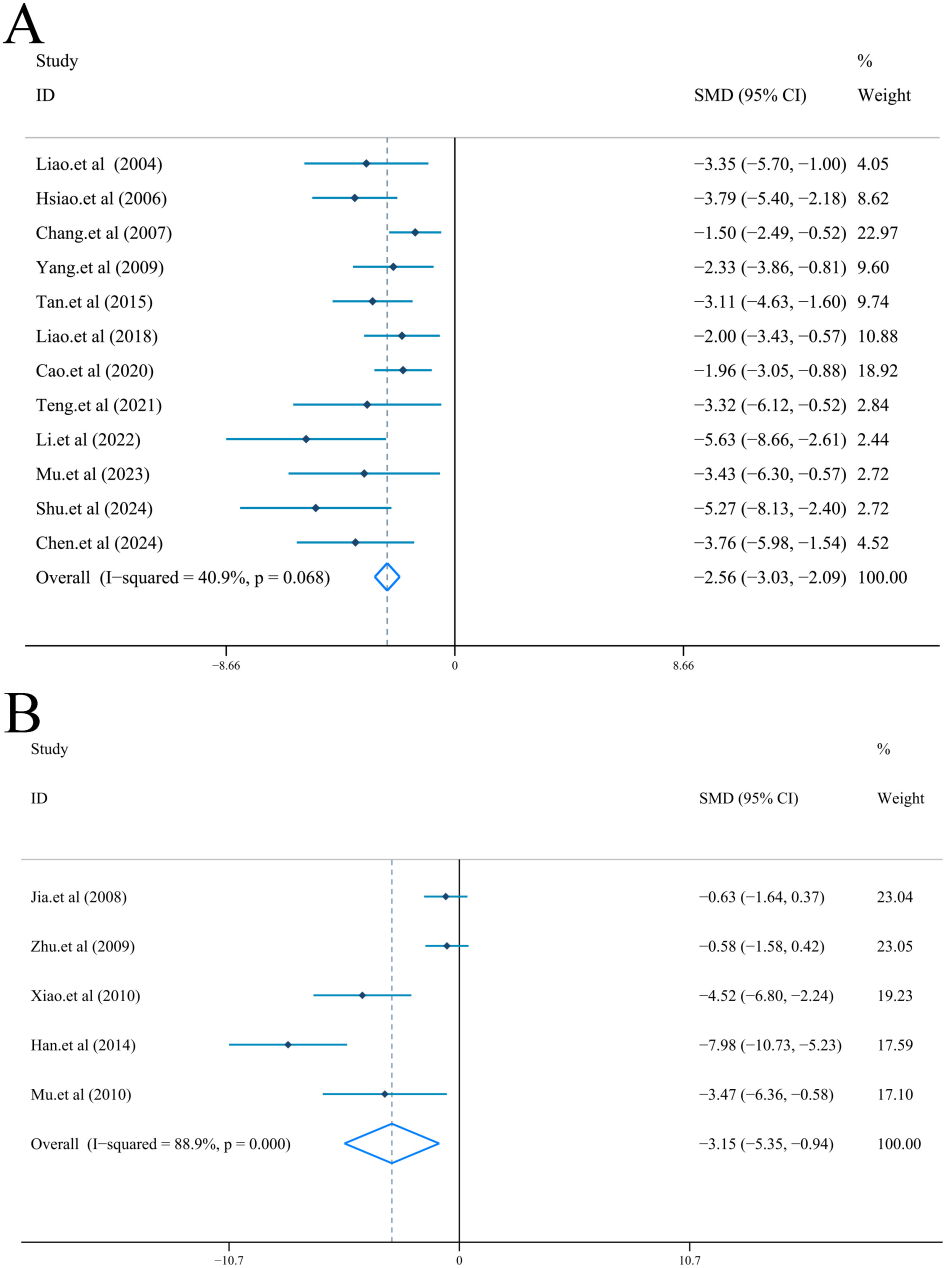
Forest plot (effect size and 95% CI) summarizing the effect of Ligustrazine on infarct volume (%) and infarct volume. **(A)** Infarct volume (%); **(B)** Infarct volume.

Fixed-effect model [*I*^2^ = 40.9%, *P* = 0.068] was used to combine the data from 12 studies (Liao et al., 2004, 2018; Hsiao et al., 2006; Chang et al., 2007; Yang et al., 2009; Tan et al., 2015; Cao et al., 2020; Teng et al., 2021; Li et al., 2022; Mu et al., 2023; Chen et al., 2024; Shu et al., 2024). Results of the quantitative merge indicated that ligustrazine effectively reduced the cerebral infarct volume (%) after cerebral I/R injury [*n* = 150, *SMD* = −2.56, *95%CI* (−3.03, −2.09), *P* < 0.001] (Figure 5A). Furthermore, subgroup analysis based on the species showed that ligustrazine was effective in reducing cerebral infarction (%) in both rats [*n* = 134, *SMD* = −2.46, *95%CI* (−2.94, −1.97), *P* < 0.001; *I*^2^ = 36.3%, *P* = 0.118] and mice [*n* = 16, *SMD* = −4.47, *95%CI* (−6.55, −2.39), *P* < 0.001; *I*^2^ = 6.7%, *P* = 0.301] (Supplementary Figure 1B). In the meanwhile, no significant publication bias was revealed by the test (|t| = 5.25>0.05, Supplementary Figure 3A). Sensitivity analysis showed that after removing any one of the 12 studies, the combined results of the remaining 11 studies were still consistent with the original pooled results (Supplementary Figure 3B).

In addition, five studies reported infarct volume (Jia et al., 2009; Zhu et al., 2009; Xiao et al., 2010; Han et al., 2014; Mu et al., 2023). Because of the high heterogeneity, this index was pooled with random-effect model [*I*^2^ = 88.9%, *P* < 0.001]. In animals with cerebral I/R injury, the infarct volume was effectively reduced by ligustrazine [*n* = 70, *SMD* = −3.15, *95%CI* (−5.35, −0.94), *P* = 0.005] (Figure 5B). At the same time, Egger's inspection revealed no significant publication bias (|t| = 3.92>0.05, Supplementary Figure 4A). Sensitivity analysis showed that after removing any one of the 5 studies, the combined results of the remaining 4 studies were still consistent with the original pooled results (Supplementary Figure 4B).

#### 3.4.4 Brain content water

**Figure 6:**
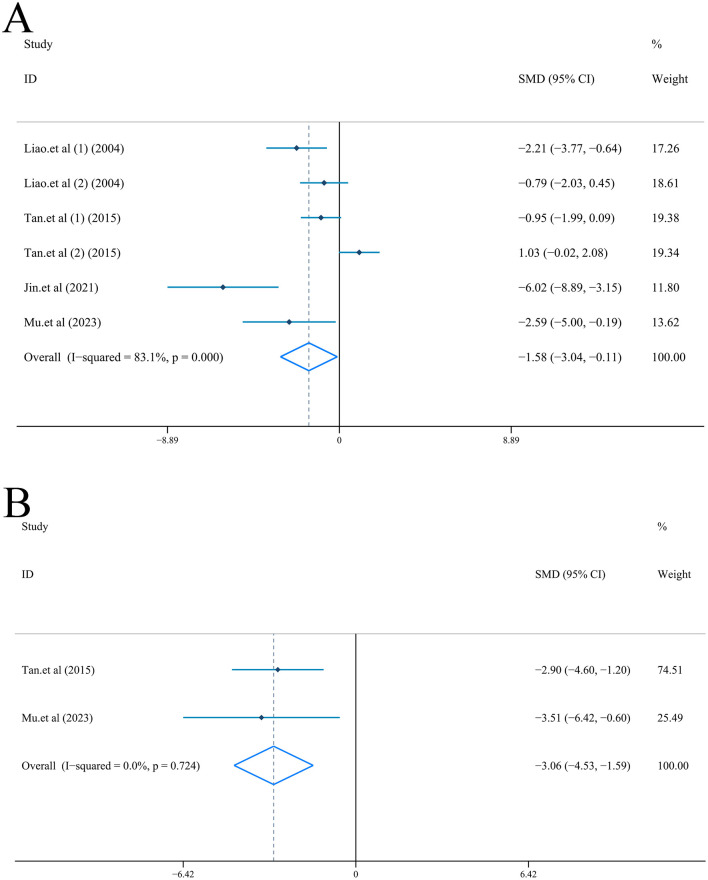
Forest plot (effect size and 95% CI) summarizing the effect of Ligustrazine on brain content water and BBB. **(A)** Brain content water; **(B)** BBB.

Random-effect model (*I*^2^ = 83.1%, *P* < 0.001) was used to combine the data from 6 studies (5, 13, 19, 20). The results revealed that ligustrazine effectively reduced the brain content water after cerebral I/R injury [*n* = 72, *SMD* = −1.58, *95%CI* (−3.04, −0.11), *P* = 0.035] (Figure 6A). Furthermore, = species of subgroup analysis showed that ligustrazine significantly reduced brain content water in mice [*n* = 18, *SMD* = −4.21, *95%CI* (−7.56, −0.86), *P* = 0.014; *I*^2^ = 68.9%, *P* = 0.073], and it could not reduce brain content water in rats [*n* = 54, *SMD* = −0.66, *95%CI* (−1.94, 0.62), *P* < 0.001; *I*^2^ = 78.0%, *P* = 0.003] (Supplementary Figure 1C). No significant publication bias was revealed by the test (|t| = 2.94>0.05, Supplementary Figure 5A). Sensitivity analysis showed that after removing any one of the 6 studies, the combined results of the remaining 5 studies were still consistent with the original pooled results (Supplementary Figure 5B).

#### 3.4.5 Permeability of blood-brain barrier

Based on the Evans blue assay, two studies reported BBB permeability (Tan et al., 2015; Mu et al., 2023). Data from 2 studies was combined by fixed-effect model (*I*^2^ = 0.0%, *P* = 0.724). For the results, ligustrazine could effectively reduce the permeability of BBB after cerebral I/R injury [*n* = 18, *SMD* = −3.06, *95%CI* (−4.53, −1.59), *P* < 0.001] (Figure 6B).

### 3.5 Secondary outcomes

#### 3.5.1 Cells in the subventricular zone

Based on the BrdU assay, cell number changes in the subventricular zone (SVZ) were reported in two studies (Qi et al., 2007a; Xiao et al., 2010). Fixed-effect model (*I*^2^ = 35.5%, *P* = 0.213) was used to combine the data from 2 articles. After cerebral I/R injury, ligustrazine could effectively promote cell-proliferation in SVZ [*n* = 24, *SMD* = 1.30, *95%CI* (0.39, 2.21), *P* = 0.005] (Supplementary Figure 6A).

#### 3.5.2 Content of MDA in cerebral cortex

Fixed-effect model (*I*^2^ = 0.0%, *P* = 0.741) was used to combine the data from 2 studies (Yang et al., 2009; Yu B. et al., 2016). The pooled results indicated that ligustrazine effectively reduced the content of MDA in brain cortex [*n* = 32, *SMD* = −2.90, *95%CI* (−3.93, −1.87), *P* < 0.001] (Supplementary Figure 6B).

#### 3.5.3 Expression of thioredoxin

Fixed-effect model (*I*^2^ = 41.8%, *P* = 0.190) was used to combine the data from 2 studies (Jia et al., 2009; Zhu et al., 2009). The pooled results indicated that ligustrazine effectively increased the expression of thioredoxin-1 (TrX-1) in brain cortex after the injury [*n* = 32, *SMD* = 4.69, *95%CI* (3.26,6.12), *P* < 0.001] (Supplementary Figure 7A).

Random-effect model (*I*^2^ = 92.9%, *P* < 0.001) was used to combine the data from 2 studies (Jia et al., 2009; Zhu et al., 2009). The pooled results indicated that ligustrazine could not effectively increased the expression of thioredoxin-2 (TrX-2) in brain cortex after the injury [*n* = 32, *SMD* = 6.31, *95%CI* (−1.71, 14.33), *P* = 0.123] (Supplementary Figure 7B).

#### 3.5.4 Expression of thioredoxin reductase

Fixed-effect model (TrXR-1, *I*^2^ = 0.0%, *P* = 0.730; TrXR-2, *I*^2^ = 0.0%, *P* = 0.921) was used to combine the data from 2 studies (Jia et al., 2009; Zhu et al., 2009). The pooled results indicated that ligustrazine could not effectively increased the expression of thioredoxin reductase 1 (TrXR-1) [*n* = 32, *SMD* = 0.58, *95%CI* (−0.13, 1.29), *P* = 0.111] (Supplementary Figure 8A) in brain cortex, while ligustrazine effectively increased the expression of thioredoxin reductase 2 (TrXR-2) [*n* = 32, *SMD* = 4.15, *95%CI* (2.86,5.44), *P* < 0.001] (Supplementary Figure 8B).

### 3.6 Mechanisms of treatment

The mechanism of ligustrazine's treatment of cerebral I/R injury was investigated, and its therapeutic effect was related to inflammatory pathways including NF-κb and JAK-STAT, and also to the regulation of oxidative stress, including TrX system and MDA content. In addition, ligustrazine regulated the contents of HIF-α, MCPIP1 and SYP to promote neurological function and reduce the permeability of BBB after injury. Supplementary Table 2 provides detailed information on the relevant mechanisms for each article.

### 3.7 Machine learning

#### 3.7.1 Sample characteristics

**Figure 7:**
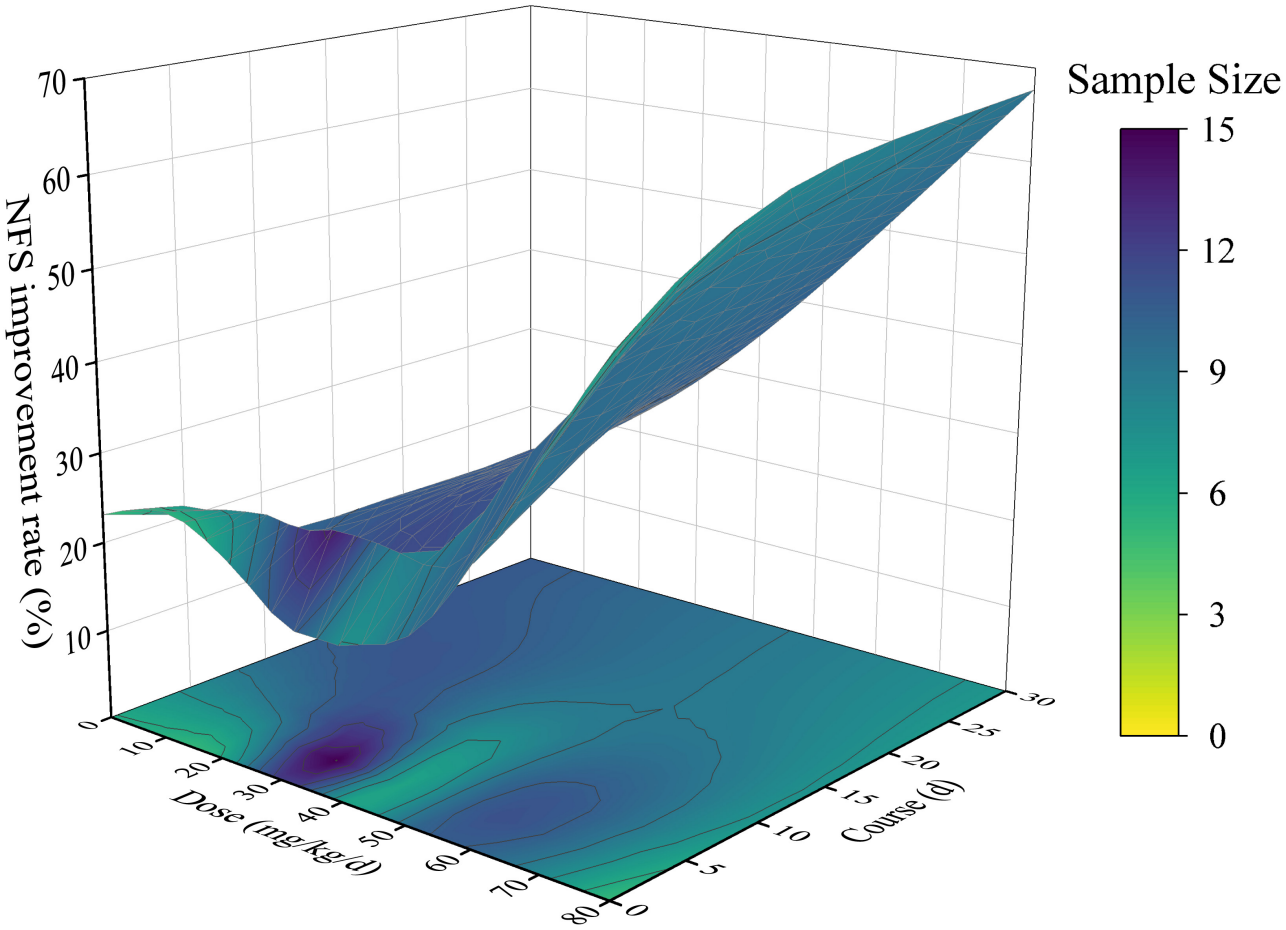
Time-dose-efficacy interval distribution with NFS as curative effect standard.

NFS of 321 samples were analyzed in this study, in which the dosage range of ligustrazine 10 mg/kg/day to 80 mg/kg/day, the duration of ligustrazine administration was extended from 2 h to 670 h, the ischemic time was 1–2 h, and the improvement rate of NFS was increased from 2.96% to 68.29% (Figure 7).

#### 3.7.2 Model development and evaluation

**Figure 8:**
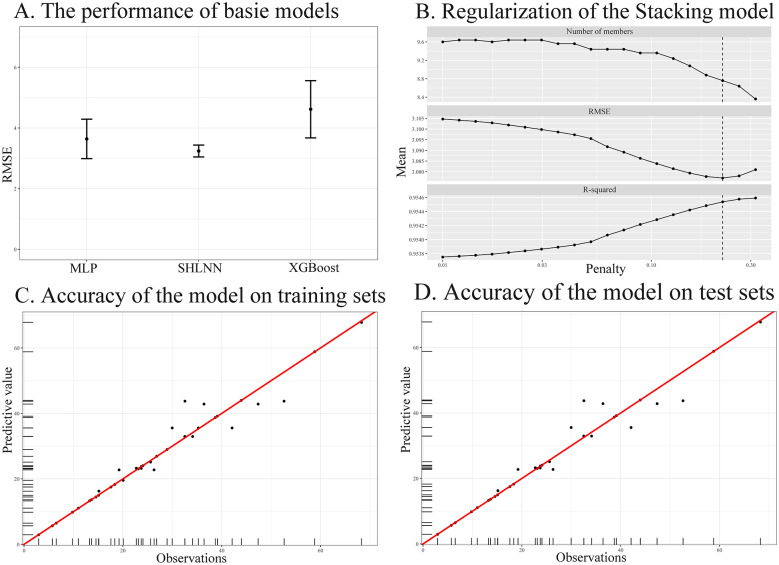
The performance of base models, regularization of the Stacking ensemble model, and the accuracy of the model on the dataset **(A)** The performance of base models. This panel shows the RMSE of three base models—MLP, SHLNN, and XGBoost—during the training process. Lower RMSE values indicate better model performance. **(B)** Regularization of the Stacking ensemble model. This panel illustrates changes in RMSE, and R^2^ as the penalty coefficient changes. The dashed line indicates the optimal regularization parameter selected for the final model. **(C)** Accuracy of the stacked model on the training sets. The horizontal axis represents the observed values, and the vertical axis represents the predicted values. The red diagonal line indicates the ideal prediction scenario. **(D)** Accuracy of the stacked model on the test sets. Similar to the training dataset, this panel evaluates the model's generalization performance on test sets.

In this study, MLP, XGBoost, and SHLNN models were utilized and further refined and enhanced through the use of a stacking algorithm. Following hyperparameter optimization, the models achieved significant accuracy, with the RMSE remaining below 4.62 in a consistent manner (Figure 8A). Subsequently, the stacked model underwent further refinement and regularization, leading to effective performance improvements (Figure 8B). During the evaluation phase, the meta-model demonstrated excellent performance metrics in both the training and test datasets: RMSE values were 2.81 and 3.58, R^2^ values were 0.961 and 0.940, and MAE values were 1.28 and 1.70, respectively (Figures 8C, D). These metrics reveal that the model possesses good efficacy and robustness.

#### 3.7.3 Model interpretation

**Figure 9:**
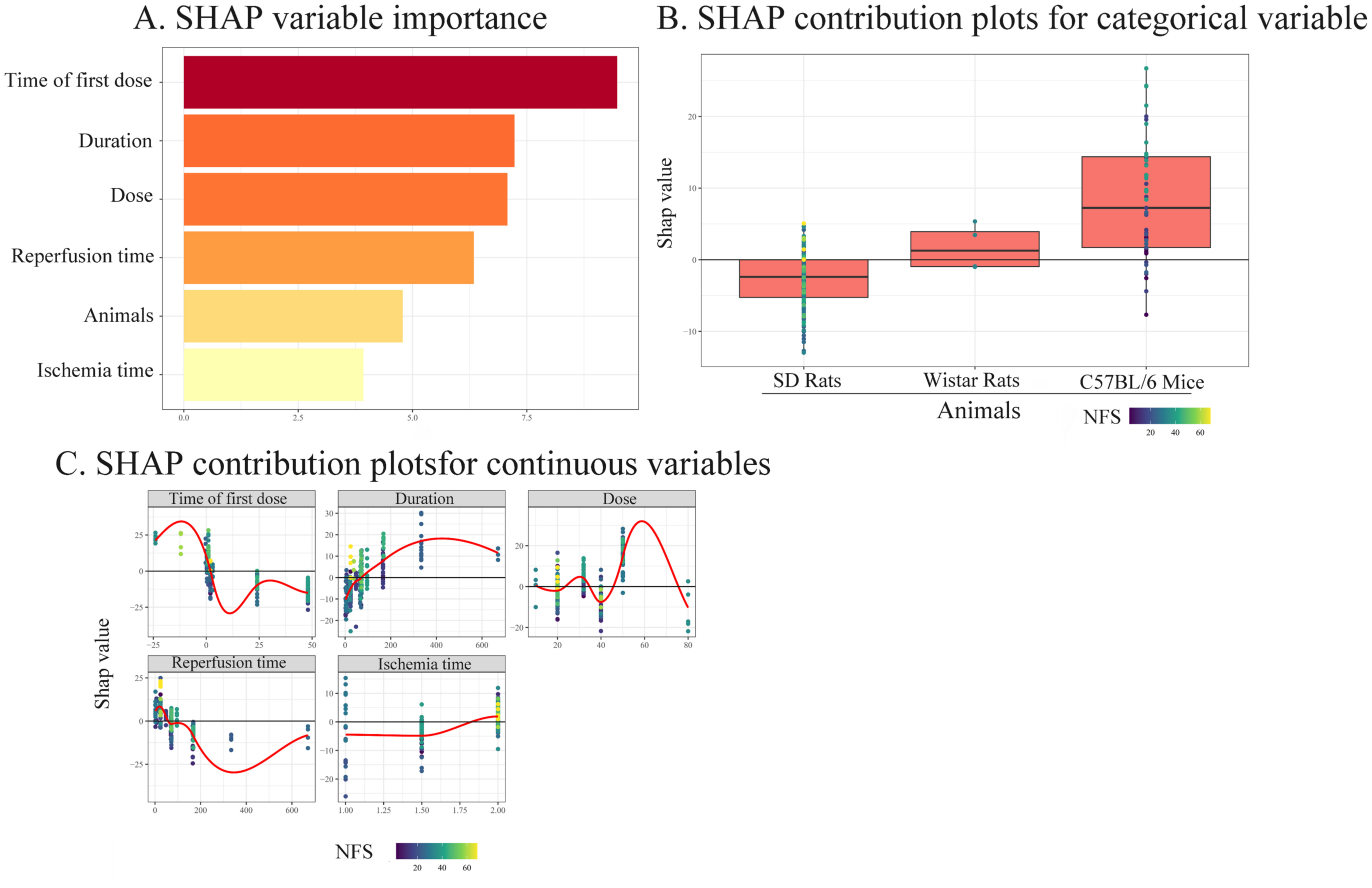
SHAP variable importance and SHAP contribution plots based on variable types **(A)** SHAP value variable importance map. The horizontal axis represents the mean contribution of each variable to the model predictions (SHAP value), and the higher SHAP values indicate a greater influence of the variable on model predictions. **(B)** SHAP contribution plot based on categorical variables. This plot illustrates the differences in the effects of different experimental animal types on the model predictions, and a positive SHAP value indicated that the category had a positive effect on the prediction results. **(C)** SHAP contribution plot based on continuous variable. This plot shows the relationships between the key continuous variables and their contributions to the model predictions (SHAP value). The red curve is used to understand the nonlinear effect of variable changes on the model prediction results. A positive SHAP value indicates that the corresponding variable may have a positive impact on the indicator improvement rate relative to the baseline value and vice versa. [**(A)** Categorical variable: the baseline value is the category with the highest frequency of occurrence in the entire training set. **(B)** Continuous variable : the baseline value is the median number of that continuous feature in the entire training set].

The Shapley value variable importance plot of the predictive model comprises six features, ranked in accordance with their impact on NFS improvement rate. The higher the absolute value, the greater the impact on NFS improvement rate. Among them, time of the first dose, duration, dose, and reperfusion time made a significant contribution to improving the NFS (Figure 9A). For categorical variable, Wistar rats and C57BL/6 mice exerted a positive effect on the improvement of NFS by Ligustrazine (Figure 9B). For continuous variables, the variables influencing NFS in descending order were the time of the first dose, duration, dose, reperfusion time, and stroke time. Figure 9C illustrates that ligustrazine had a positively affected NFS in animals with cerebral I/R injury when the time of first dose was within 24 h prior to modeling (and within 2.21 h post-modeling), the doses ranged from 23.53 to 34.69 mg/kg/d (and from 45.71 to 75.65 mg/kg/d), the medication duration exceeded 71.43 h, reperfusion time was less than 58.25 h, and ischemic time was 1.80–2.00 h. These ranges represent model-derived associations and should be interpreted as exploratory findings rather than definitive optimal intervention parameters.

## 4 Discussion

### 4.1 Aggregation of the evidence

A meta-analysis and systematic review of 23 preclinical articles (381 animals) demonstrated the potential beneficial efficacy of ligustrazine in treating cerebral I/R injury. According to the meta-analysis findings, the primary benefit of ligustrazine was observed in the improvement of inflammation indicators (COX-2, IL-6, IL-1β, TNF-α, etc.), oxidative stress indicators (SOD, GSH-Px, MDA, etc.), apoptosis indicators such as caspase-3, promoted neuronal cell proliferation, and reduced permeability of BBB after cerebral I/R injury. The potential mechanisms underlying these protective effects may be associated with the impact of ligustrazine on promoting angiogenesis, neuronal nutrition, anti-inflammation, anti-oxidation and anti-apoptosis. While the traditional meta-analysis demonstrated the overall efficacy of ligustrazine, it was unable to accurately predict key intervention parameters such as dose, time of the first dose, and treatment duration. To address this limitation, machine learning approaches were employed. Using NFS as the dependent variable, predictive models were constructed using a 7.5:2.5 training-to-test data ratio and validated through 5-fold cross-validation to ensure generalizability and prevent overfitting. The machine learning analysis identified the time of the first dose, dosage, and duration of administration as critical determinants of therapeutic efficacy. Specifically, enhanced neurofunctional recovery was observed when ligustrazine was administered within 24 h prior to, or approximately 2.21 h following, ischemia onset, at doses ranging from 23.53 to 34.69 mg/kg/day (or 45.71–75.65 mg/kg/day), and sustained for more than 71.43 h. These quantitative thresholds establish a data-driven foundation for optimizing preclinical dosing regimens of ligustrazine.

Integrating the findings from both meta-analysis and machine learning, this study not only substantiates the broad-spectrum neuroprotective efficacy of ligustrazine in cerebral I/R injury but also establishes a robust preclinical evidence framework that elucidates the mechanistic underpinnings and key intervention parameters. This framework facilitates the rational design of treatment protocols with precise timing, dosage, and duration, thereby enhancing the potential for successful translational research and clinical application in stroke and related cerebrovascular disorders.

### 4.2 Potential mechanism of action

**Figure 10:**
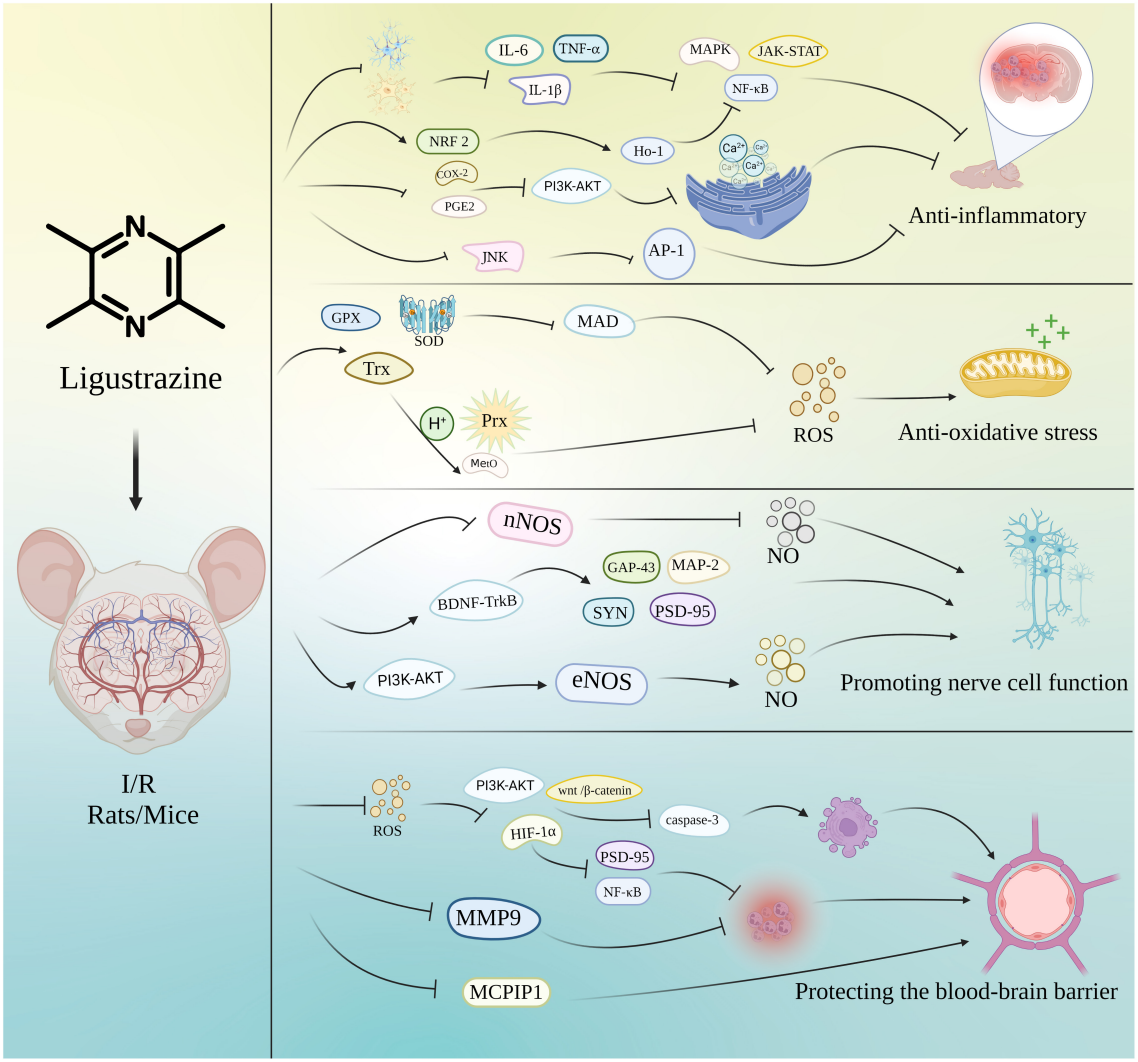
Mechanisms of ligustrazine in the treatment of cerebral I/R injury.

The review of the included studies revealed that the main mechanisms underlying the therapeutic effects of ligustrazine included the following (Figure 10).

#### 4.2.1 Anti-inflammatory

Activation of the inflammatory cascade response is closely associated with neurological dysfunction in ischemic region after cerebral I/R injury (Shi et al., 2019). The inflammatory response in the ischemic region primarily occurs through the activation of glial cells in the brain (Guruswamy and ElAli, 2017). When exposed to ischemia and hypoxia conditions, activated astrocytes and polarized M1-type microglia produce stress pro-inflammatory cytokines, such as IL-1β, IL-6, TNF-α, etc., which were then triggered to start to be upregulated within the first few hours of the acute response (Taylor and Sansing, 2013; Burda et al., 2016). Meanwhile, the released inflammatory factors will recruit peripheral immune cells to ischemic hemisphere, causing infiltration of local brain tissue inflammatory cells, especially neutrophils, which positively affects the associated immune response after ischemic injury (Taylor and Sansing, 2013).

When inflammation occurs in tissues, COX-2 induced in the body by inflammatory mediators, exerting various pro-inflammatory effects (Simon, 1999). COX-2 can promote the production of PEG2, which plays a pivotal role in initiating the subsequent inflammatory response, including enhancing the movement of immune cells to the sites of inflammation (Park et al., 2006).

Animals under the MCAO I/R model showed remarkable glial activation, accompanied by the release of inflammatory factors, while interventional treatment of ligustrazine significantly reduced the degree of microglial and astrocyte activation (Kao et al., 2013; Jin et al., 2021), and the levels of inflammatory factors also produced a corresponding reduction (Chang et al., 2007; Yu B. et al., 2016). In addition, ligustrazine also reduced the pro-inflammatory mediators levels including COX-2 and PGE2 after I/R injury and relieved the peripheral immune cells infiltration in ischemic hemisphere (Liao et al., 2004; Yang et al., 2009). The manifestations of these series reflect the overall inhibition of ligustrazine inflammatory response in the brain of stroke rodents, and exerted a significant anti-inflammatory effect.

Activation of inflammatory factors can trigger the transduction of a range of intracellular signaling pathways, including NF-κB, JNK/AP-1, and JAK-STAT signaling pathway, etc. (Ye, 2015; Mitchell et al., 2016; Banerjee et al., 2017). Among these, NF-κB pathway as the classical regulatory pathway related to inflammation, and NRF 2, a core protein that regulates the transcription of antioxidant proteins, has been proved by many studies to have multiple direct or indirect effects with NF-κB pathway, affecting the process of inflammatory response (Keleku-Lukwete et al., 2018). As a mitogen-activated protein kinase, JNK is a key target in the inflammatory response. The activation of JNK could induce inflammatory cells to release cytokines and chemokines, and continuously stimulates the activation of the AP-1 transcription factor., which further induced the inflammatory progression (Vukic et al., 2009). In related studies, the use of ligustrazine promoted the activation of NRF 2 and its downstream antioxidant enzyme HO-1 in I/R rodents, in the meanwhile, ligustrazine inhibited the transcription level of JNK and AP-1(Kao et al., 2013; Chang et al., 2015), these function may be the key mechanism for ligustrazine to exert its anti-inflammatory effect.

#### 4.2.2 Anti-oxidative stress

Oxidative stress is recognized as a primary mechanism of action in the context of ischemic stroke. Studies have shown that ROS, which are produced in large amounts during acute hypoxia and reperfusion (McGarry et al., 2018), can cause peroxidation of proteins, DNA, and lipids, producing oxidative stress, being the principal contributor to the majority of secondary injuries (Fukai and Ushio-Fukai, 2011). SOD, catalase and glutathione reduction system are the main systems for scavenging superoxide *in vivo* (Fukai and Ushio-Fukai, 2011; Ribas et al., 2014), can scavenge excessive superoxide *in vivo* under physiological conditions and maintain the balance between oxidation and antioxidation, while the excessive production of ROS exacerbates oxidative stress in cells, causing impaired mitochondrial function and leading to a disruption of the intrinsic antioxidant system's capacity, causing different cascades of cell impaired antioxidant function (Jones, 2008). However, ligustrazine can improve cerebral I/R injury by regulating mitochondrial dynamics (Chen et al., 2024).

TrX is a key antioxidant protein of the thioredoxin system for defensing against oxidative stress (Andoh et al., 2002). Trx exerts its antioxidant effect is the delivery of its own strongly reducing hydrogen atoms to enzymes involved in various reduction reactions, such as ribonucleotide reductase, peroxiredoxin, methionine sulfoxide reductase, etc. (Cunningham et al., 2015). Using ligustrazine significantly reduced ROS, increased the activities of GPX and other antioxidant enzymes, and significantly increased the expression of TrX (Chen et al., 2024). In the meanwhile, ligustrazine reduced the level of MDA *in vivo*. The present studies suggested that ligustrazine enhanced the antioxidant stress ability of animals (Jia et al., 2009; Zhu et al., 2009; Yu B. et al., 2016).

#### 4.2.3 Promoting nerve cell function

The repair of neuronal cell proliferation and synaptic plasticity that occurs in brain injury diseases is an important mechanism for functional brain repair (Ni et al., 2020). NO is a free radical gas that is involved in various biological processes in the CNS, where it can act as a neurotransmitter and regulate neural function (Cinelli et al., 2020). Among the NO-producing NOS family, there are three isoforms, iNOS, nNOS and eNOS. As an important neurotransmitter and regulator, NO has a bidirectional effect on the regulation of the nervous system. NO can maintain normal communication between neurons and synaptic remodeling and confers a neuroprotective effect under physiological conditions (Hardingham et al., 2013), while excessive NO, which is highly oxidizing and nitrifying, can rapidly oxidize glucose and lipids to nitrate and nitrite (Burney et al., 1999), exerting an inhibitory effect on neuronal cell proliferation (Calabrese et al., 2007). Furthermore, related studies show that NO produced in Neurons-Derived nNOS (Michel and Feron, 1997), can negatively affect the neurogenesis (Zhang et al., 2012). However, NO derived from eNOS appears to have neuroprotective effects (Garry et al., 2015). The application of ligustrazine can inhibit the activity of nNOS and promote the proliferation of neuronal cells, while it also increased the level of beneficial eNOS, by activating PI3K/Akt pathway, playing a neuron-protective role (Qi et al., 2007a; Ding et al., 2019). BNDF is an important nerve growth factor, and activation of BDNF-TrkB signaling is an important signaling pathway for neuronal growth, genesis and survival, and its mechanisms of action include regulation of synaptic structure and branching, enhancement of synaptic plasticity, and regulation of neurotransmitters (Andero et al., 2014). Among them, the related marker proteins of synaptic plasticity mainly include: MAP-2, GAP-43, SYN, PSD-95, etc. These proteins play a vital role in the development and formation of synapses, promoting enhanced synaptic function (Sadigh-Eteghad et al., 2018). In addition, angiogenesis is essential for the recovery of cerebral blood flow and the maintenance of nerve cell function. Ligustrazine can alleviate cerebral I/R injury by promoting angiogenesis (Shu et al., 2024).

Ligustrazine increased BrdU positive cells, suggested it has the ability to stimulate the proliferation of neural stem cells, probably by upregulating the BDNF related pathway, which exerts the function of nourishing nerve cells (Xiao et al., 2010; Chen et al., 2024). The results of Synaptic Ultrastructures revealed a less curved synaptic interface and a wider synaptic in ligustrazine treatment group I/R rodents, and the increased protein content of MAP-2, GAP-43, SYN, PSD-95 showed that it promoted prominent repair and reconstruction of neural pathways (Lin et al., 2015, 2021).

#### 4.2.4 Protecting BBB

Damage to BBB is one of the crucial pathogenic mechanisms of ischemic stroke (Jiang et al., 2018). Brain microvascular endothelial cells, astrocytes, pericytes and the tight junctions between them constitute the BBB. Altered permeability of the BBB is closely associated with numerous biological activities, including inflammation, oxidative stress, and apoptosis. In the inflammatory cascade response, endothelial cells are activated to produce a series of pro-inflammatory factors, and the infiltration of inflammatory factors causes a vascular response and a decrease in BBB permeability, further enhancing the infiltration of peripheral inflammatory cells (Shi et al., 2019). ligustrazine not only can inhibit inflammation in rodents by reducing the secretion of inflammatory mediators, new studies show that MCPIP1, a newly discovered zinc-finger protein, is reduced during ligustrazine treatment of I/R animals and further protects BBB permeability (Jin et al., 2021).

MMP9, a critical member of the protein hydrolase family, is generated with the help of neutrophils and can play a role in degrading extracellular matrix components (Wells et al., 2015), and its activation significantly causes damage to the cellular matrix and basal lamina, further promoting inflammatory and immune responses, causing damage to the BBB and exacerbating the worsening outcome of stroke. Evaluation of rodent BBB permeability changes by Evans blue staining and FITC method showed a significant increase of BBB permeability in I/R rodents and disruption of tight junction proteins, accompanied by increased MMP-9 protein levels. Treatment with ligustrazine significantly inhibited this disruption of BBB function, acted as a certain repair of tight junction proteins, inhibited the activation of MMP-9, and avoided its subsequent effects (Tan et al., 2015; Jin et al., 2021).

The production of various imbalanced peroxides as well as superoxide in oxidative stress is highly reactive and can directly oxidize biomolecules, causing direct damage to BBB structure (Chen et al., 2022), while signaling molecules of oxidative stress, such as ROS, can cause direct damage to BBB structure by regulating such pathways as PI3K-AKT, HIF-1α, Wnt/β-catenin and other interconnected stress-responding signaling pathways including cellular autophagy, apoptosis, iron death, etc. (Bhatia and Sharma, 2021; Janbandhu et al., 2022; Wang et al., 2022), affecting cell survival and thus causing disruption of BBB function and structure.

Among them, HIF-1α is an important receptor and effector regulated by oxygen, an important regulator of oxygen homeostasis, and regulates the cellular response to the stressful environment and mediates the transcription of several genes (Kallio et al., 1999). Upregulation of the HIF-1α-VEGF axis exacerbates blood-brain barrier permeability (Chen et al., 2010), further triggers post-hypoxic injury. Sustained stimulation of HIF-1α increases intracellular caspase-3 protein levels, which promotes apoptosis (Zhang et al., 2012), resulting in BBB destruction. In addition, HIF-1α mediates the inflammatory response through with NF-κB, Notch signaling pathway (Bruning et al., 2012; Gao et al., 2012), exacerbating the leakage of BBB. Several studies have shown that increased levels of HIF1α are detected in several cerebral cells during stroke and verified that it has a deleterious effect, involved in including inflammatory responses, apoptosis, and autophagy. Ligustrazine reduced HIF-1α levels (Chang et al., 2007), indicating that HIF-1α may serve as a key target for ligustrazine to exert relevant anti-inflammatory and antioxidant effects, and is closely related to improve BBB function. The above-mentioned related mechanisms suggest that in ischemic stroke, the impairment of the BBB is one of the outcomes of various biological processes crosstalk. Related studies have explored and confirmed the intervention effect of ligustrazine on crosstalk from different perspectives of these related mechanisms, effectively protected the BBB function, playing an important therapeutic role (Jin et al., 2021; Mu et al., 2023).

### 4.3 Limitations

This quantitative analysis summarized the existing evidence and the related mechanisms. Although the study was pre-registered for the implementation of the protocol, there were still some limitations due to the preclinical meta-analysis, which are as follows:

(a) In animal studies, negative results were less likely to be accepted than positive results, which may lead to some bias.
(b) These studies have some methodological shortcomings, and none of them reported blinded induction of cerebral I/R injury.
(c) Machine learning models exhibit high interpretability; however, small samples may affect the accuracy of feature selection, meanwhile, there is a lack of external data sets for further validation.
(d) Each of the models included had some shortcomings, such as the limited ability of SHLNN to deal with complex data. Although through data standardization to simplify the data set, and the limitation of some single models was reduced by using Stacking algorithm to integrate these models, it also increases the overall complexity of the model, and put forward the challenge of interpretability.
(e) First, potential sex bias may exist because most preclinical studies predominantly used male animals, which may limit the generalizability of the findings. Second, although subgroup analyses and sensitivity analyses were conducted to explore possible sources of heterogeneity and to assess the robustness of the results, relatively high heterogeneity was observed in some outcomes. Third, although publication bias was assessed statistically, the possibility of selective reporting cannot be entirely excluded, as studies with positive findings are more likely to be published. Finally, the present analysis is mainly based on preclinical evidence. Due to interspecies differences in pharmacokinetics and pathophysiology, caution should be exercised when translating these findings into clinical practice. Therefore, more rigorously designed studies with standardized methodologies, as well as well-designed clinical trials, are needed to further validate the therapeutic potential of ligustrazine.

### 4.4 Implications

Cerebral I/R injury is a multifaceted level involving many factors and mechanisms. Ligustrazine, as a natural monomer, has attracted much attention because it improves hemodynamics during cerebral I/R injury. Consequently, an increasing number of relevant animal studies have been conducted. In the present study, meta-analytic results indicated that ligustrazine could significantly improve NFS, reduce the cerebral infarct volume, and improve the integrity of BBB in experimental animals, thereby exerting a therapeutic effect in cerebral I/R injury via diverse mechanisms and pathways. These findings were consistent with the results of previous studies, and further substantiating the neuroprotective potential of ligustrazine. Notably, machine learning analysis based on the NFS improvement rate revealed the key influencing factors of ligustrazine's efficacy, including the time of first dose, duration, and dose, and further determined the optimal dosage course, which provided a basis for future experiments and accords with experimental ethics more. In summary, this research leveraged advanced statistical method and machine learning to provide comprehensive and robust scientific evidence for the treatment of ligustrazine in cerebral I/R injury, offering novel insights for subsequent investigations. In the future, we will further explore the role of ligustrazine in different disease models, aiming to inform therapeutic strategies and options for cerebral ischemic conditions.

## 5 Conclusion

Based on meta-analysis and machine learning, this study identified the significant potential of ligustrazine in the treatment of cerebral I/R injury. Notably, the model suggested that greater improvements in neurological function were associated with certain ranges of intervention parameters, includingthe time of first dose was within 24 h prior to modeling (and within 2.21 h post-modeling), the doses ranged from 23.53 to 34.69 mg/kg/d (and from 45.71 to 75.65 mg/kg/d), and the medication duration exceeded 71.43 h. However, aiming to accurately evaluate the therapeutic role and safety of ligustrazine in I/R injury, additional targeted and high-quality preclinical studies required to consolidate the evidence chain.

## References

[B1] Andero R. Choi D. C. Ressler K. J. (2014). “Chapter six - BDNF–TrkB receptor regulation of distributed adult neural plasticity, memory formation, and psychiatric disorders,” in Progress in Molecular Biology and Translational Science, eds. Z. U. Khan and E. C. Muly (Cambridge, MA: Academic Press), 169–192. 10.1016/B978-0-12-420170-5.00006-4 24484701

[B2] Andoh T. Chock P. B. Chiueh C. C. (2002). The roles of thioredoxin in protection against oxidative stress-induced apoptosis in SH-SY5Y cells. J. Biol. Chem. 277, 9655–9660. doi: 10.1074/jbc.M110701200 11751890

[B3] Banerjee S. Biehl A. Gadina M. Hasni S. Schwartz D. M. (2017). JAK-STAT signaling as a target for inflammatory and autoimmune diseases: current and future prospects. Drugs 77, 521–546. doi: 10.1007/s40265-017-0701-9 28255960 PMC7102286

[B4] Bhatia V. Sharma S. (2021). Role of mitochondrial dysfunction, oxidative stress and autophagy in progression of Alzheimer's disease. J. Neurol. Sci. 421:117253. doi: 10.1016/j.jns.2020.117253 33476985

[B5] Bruning U. Fitzpatrick S. F. Frank T. Birtwistle M. Taylor C. T. Cheong A. (2012). NFκB and HIF display synergistic behaviour during hypoxic inflammation. Cell. Mol. Life Sci. 69, 1319–1329. doi: 10.1007/s00018-011-0876-2 22068612 PMC11114791

[B6] Burda J. E. Bernstein A. M. Sofroniew M. V. (2016). Astrocyte roles in traumatic brain injury. Exp. Neurol. 275, 305–315. doi: 10.1016/j.expneurol.2015.03.020 25828533 PMC4586307

[B7] Burney S. Caulfield J. L. Niles J. C. Wishnok J. S. Tannenbaum S. R. (1999). The chemistry of DNA damage from nitric oxide and peroxynitrite. Mutat. Res. 424, 37–49. doi: 10.1016/S0027-5107(99)00006-8 10064848

[B8] Calabrese V. Mancuso C. Calvani M. Rizzarelli E. Butterfield D. A. Stella A. M. G. (2007). Nitric oxide in the central nervous system: neuroprotection versus neurotoxicity. Nat. Rev. Neurosci. 8, 766–775. doi: 10.1038/nrn2214 17882254

[B9] Cao H. Cheng Y. Zhang J. Xu M. Ge L. (2020). The effect of umbilical cord mesenchymal stem cells combined with tetramethylpyrazine therapy on ischemic brain injury: a histological study. J. Stroke Cerebrovasc. Dis. 29:105298. doi: 10.1016/j.jstrokecerebrovasdis.2020.105298 32992203

[B10] Chang C.-Y. Kao T.-K. Chen W.-Y. Ou Y.-C. Li J.-R. Liao S.-L. . (2015). Tetramethylpyrazine inhibits neutrophil activation following permanent cerebral ischemia in rats. Biochem. Biophys. Res. Commun. 463, 421–427. doi: 10.1016/j.bbrc.2015.05.088 26043690

[B11] Chang Y. Hsiao G. Chen S. Chen Y. Lin J. Lin K. . (2007). Tetramethylpyrazine suppresses HIF-1α, TNF-α, and activated caspase-3 expression in middle cerebral artery occlusion-induced brain ischemia in rats. Acta Pharmacol. Sin. 28, 327–333. doi: 10.1111/j.1745-7254.2007.00514.x 17302993

[B12] Chen C. Ostrowski R. P. Zhou C. Tang J. Zhang J. H. (2010). Suppression of hypoxia-inducible factor-1α and its downstream genes reduces acute hyperglycemia-enhanced hemorrhagic transformation in a rat model of cerebral ischemia. J. Neurosci. Res. 88, 2046–2055. doi: 10.1002/jnr.22361 20155812

[B13] Chen S. Li L. Peng C. Bian C. Ocak P. E. Zhang J. H. . (2022). Targeting oxidative stress and inflammatory response for blood–brain barrier protection in intracerebral hemorrhage. Antioxid. Redox Signal. 37, 115–134. doi: 10.1089/ars.2021.0072 35383484

[B14] Chen X. Yang T. Zhou Y. Mei Z. Zhang W. (2024). Astragaloside IV combined with ligustrazine ameliorates abnormal mitochondrial dynamics via Drp1 SUMO / deSUMOylation in cerebral ischemia–reperfusion injury. CNS Neurosci. Ther. 30:e14725. doi: 10.1111/cns.14725 38615367 PMC11016344

[B15] Cinelli M. A. Do H. T. Miley G. P. Silverman R. B. (2020). Inducible nitric oxide synthase: regulation, structure, and inhibition. Med. Res. Rev. 40, 158–189. doi: 10.1002/med.21599 31192483 PMC6908786

[B16] Cunningham G. M. Roman M. G. Flores L. C. Hubbard G. B. Salmon A. B. Zhang Y. . (2015). The paradoxical role of thioredoxin on oxidative stress and aging. Arch. Biochem. Biophys. 576, 32–38. doi: 10.1016/j.abb.2015.02.025 25726727

[B17] De Livera A. M. Sysi-Aho M. Jacob L. Gagnon-Bartsch J. A. Castillo S. Simpson J. A. . (2015). Statistical methods for handling unwanted variation in metabolomics data. Anal. Chem. 87, 3606–3615. doi: 10.1021/ac502439y 25692814 PMC4544854

[B18] Ding Y. Du J. Cui F. Chen L. Li K. (2019). The protective effect of ligustrazine on rats with cerebral ischemia–reperfusion injury via activating PI3K/Akt pathway. Hum. Exp. Toxicol. 38, 1168–1177. doi: 10.1177/0960327119851260 31250662

[B19] Feigin V. L. Norrving B. Mensah G. A. (2017). Global burden of stroke. Circ. Res. 120, 439–448. doi: 10.1161/CIRCRESAHA.116.308413 28154096

[B20] Fukai T. Ushio-Fukai M. (2011). Superoxide dismutases: role in redox signaling, vascular function, and diseases. Antioxid. Redox Signal. 15, 1583–1606. doi: 10.1089/ars.2011.3999 21473702 PMC3151424

[B21] Gao H. Liu P. Li P. Huang Z. Yu F. Lei T. . (2015). Ligustrazine monomer against cerebral ischemia-reperfusion injury. Neural Regen. Res. 10:832. doi: 10.4103/1673-5374.156991 26109963 PMC4468780

[B22] Gao W. Sweeney C. Connolly M. Kennedy A. Ng C. T. McCormick J. . (2012). Notch-1 mediates hypoxia-induced angiogenesis in rheumatoid arthritis. Arthritis Rheum. 64, 2104–2113. doi: 10.1002/art.34397 22275240

[B23] Garry P. S. Ezra M. Rowland M. J. Westbrook J. Pattinson K. T. S. (2015). The role of the nitric oxide pathway in brain injury and its treatment — from bench to bedside. Exp. Neurol. 263, 235–243. doi: 10.1016/j.expneurol.2014.10.017 25447937

[B24] Guo X. Qin Y. Feng Z. Li H. Yang J. Su K. . (2024). Investigating the anti-inflammatory effects of icariin: a combined meta-analysis and machine learning study. Heliyon 10:e35307. doi: 10.1016/j.heliyon.2024.e35307 39170422 PMC11336647

[B25] Guruswamy R. ElAli A. (2017). Complex roles of microglial cells in ischemic stroke pathobiology: new insights and future directions. Int. J. Mol. Sci. 18:496. doi: 10.3390/ijms18030496 28245599 PMC5372512

[B26] Han J. Wan H.-T. Yang J.-H. Zhang Y.-Y. Ge L.-J. Bie X.-D. (2014). Effect of ligustrazine on levels of amino acid neurotransmitters in rat striatum after cerebral ischemia-reperfusion injury. J. Asian Nat. Prod. Res. 16, 1060–1067. doi: 10.1080/10286020.2014.935347 25159498

[B27] Hardingham N. Dachtler J. Fox K. (2013). The role of nitric oxide in pre-synaptic plasticity and homeostasis. Front. Cell. Neurosci. 7:190. doi: 10.3389/fncel.2013.00190 24198758 PMC3813972

[B28] Hsiao G. Chen Y.-C. Lin J.-H. Lin K.-H. Chou D.-S. Lin C.-H. . (2006). Inhibitory mechanisms of tetramethylpyrazine in middle cerebral artery occlusion (MCAO)-induced focal cerebral ischemia in rats. Planta Med. 72, 411–417. doi: 10.1055/s-2005-917242 16557454

[B29] Hu Q. Jiang L. Yan Q. Zeng J. Ma X. Zhao Y. (2023). A natural products solution to diabetic nephropathy therapy. Pharmacol. Ther. 241:108314. doi: 10.1016/j.pharmthera.2022.108314 36427568

[B30] Huang J. Chen L. Yao Z. Sun X. Tong X. Dong S. (2023). The role of mitochondrial dynamics in cerebral ischemia-reperfusion injury. Biomed. Pharmacother. 162:114671. doi: 10.1016/j.biopha.2023.114671 37037094

[B31] Janbandhu V. Tallapragada V. Patrick R. Li Y. Abeygunawardena D. Humphreys D. T. . (2022). Hif-1a suppresses ROS-induced proliferation of cardiac fibroblasts following myocardial infarction. Cell Stem Cell 29, 281–297.e12. doi: 10.1016/j.stem.2021.10.009 34762860 PMC9021927

[B32] Jia J. Zhang X. Hu Y.-S. Wu Y. Wang Q.-Z. Li N.-N. . (2009). Protective effect of tetraethyl pyrazine against focal cerebral ischemia/reperfusion injury in rats: therapeutic time window and its mechanism. Thromb. Res. 123, 727–730. doi: 10.1016/j.thromres.2008.11.004 19128823

[B33] Jiang X. Andjelkovic A. V. Zhu L. Yang T. Bennett M. V. L. Chen J. . (2018). Blood-brain barrier dysfunction and recovery after ischemic stroke. Prog. Neurobiol. 163–164, 144–171. doi: 10.1016/j.pneurobio.2017.10.001 PMC5886838 28987927

[B34] Jin Z. Liang J. Kolattukudy P. E. (2021). Tetramethylpyrazine preserves the integrity of blood-brain barrier associated with upregulation of MCPIP1 in a murine model of focal ischemic stroke. Front. Pharmacol. 12:710358. doi: 10.3389/fphar.2021.710358 34393790 PMC8355423

[B35] Jones D. P. (2008). Radical-free biology of oxidative stress. Am. J. Physiol. Cell Physiol. 295, C849–C868. doi: 10.1152/ajpcell.00283.2008 18684987 PMC2575825

[B36] Jurcau A. Simion A. (2021). Neuroinflammation in cerebral ischemia and ischemia/reperfusion injuries: from pathophysiology to therapeutic strategies. Int. J. Mol. Sci. 23:14. doi: 10.3390/ijms23010014 35008440 PMC8744548

[B37] Kallio P. J. Wilson W. J. O'Brien S. Makino Y. Poellinger L. (1999). Regulation of the hypoxia-inducible transcription factor 1α by the ubiquitin-proteasome pathway. J. Biol. Chem. 274, 6519–6525. doi: 10.1074/jbc.274.10.6519 10037745

[B38] Kao T.-K. Chang C.-Y. Ou Y.-C. Chen W.-Y. Kuan Y.-H. Pan H.-C. . (2013). Tetramethylpyrazine reduces cellular inflammatory response following permanent focal cerebral ischemia in rats. Exp. Neurol. 247, 188–201. doi: 10.1016/j.expneurol.2013.04.010 23644042

[B39] Katan M. Luft A. (2018). Global burden of stroke. Semin. Neurol. 38, 208–211. doi: 10.1055/s-0038-1649503 29791947

[B40] Keleku-Lukwete N. Suzuki M. Yamamoto M. (2018). An overview of the advantages of KEAP1-NRF2 system activation during inflammatory disease treatment. Antioxid. Redox Signal. 29, 1746–1755. doi: 10.1089/ars.2017.7358 28899203

[B41] Lee D. D. Luxburg U. von, Garnett, R. Sugiyama M. Guyon I. Neural Information Processing Systems Foundation eds. (2017). Advances in Neural Information Processing Systems 29: 30th Annual Conference on Neural Information Processing Systems 2016: Barcelona, Spain, 5-10 December 2016. Red Hook, NY: Curran Associates, Inc.

[B42] Lee D. K. In J. Lee S. (2015). Standard deviation and standard error of the mean. Korean J. Anesthesiol. 68:220. doi: 10.4097/kjae.2015.68.3.220 26045923 PMC4452664

[B43] Li L. Zhang D. Yao W. Wu Z. Cheng J. Ji Y. . (2022). Ligustrazine exerts neuroprotective effects via circ_0008146/miR-709/Cx3cr1 axis to inhibit cell apoptosis and inflammation after cerebral ischemia/reperfusion injury. Brain Res. Bull. 190, 244–255. doi: 10.1016/j.brainresbull.2022.10.011 36244580

[B44] Liao S.-L. Kao T.-K. Chen W.-Y. Lin Y.-S. Chen S.-Y. Raung S.-L. . (2004). Tetramethylpyrazine reduces ischemic brain injury in rats. Neurosci. Lett. 372, 40–45. doi: 10.1016/j.neulet.2004.09.013 15531085

[B45] Liao W. Huang X. Yin Y. Liu B. Zhu R. (2018). In vivo microdialysis with ultra performance liquid chromatography-mass spectrometry for analysis of tetramethylpyrazine and its interaction with borneol in rat brain and blood. Biomed. Chromatogr. 32:e4210. doi: 10.1002/bmc.4210 29431191

[B46] Lin J. Hao C. Gong Y. Zhang Y. Li Y. Feng Z. . (2021). Effect of tetramethylpyrazine on neuroplasticity after transient focal cerebral ischemia reperfusion in rats. Evid. Based Complement. Alternat. Med. 2021:1587241. doi: 10.1155/2021/1587241 33531914 PMC7834793

[B47] Lin J.-B. Zheng C.-J. Zhang X. Chen J. Liao W.-J. Wan Q. (2015). Effects of tetramethylpyrazine on functional recovery and neuronal dendritic plasticity after experimental stroke. Evid. Based Complement. Alternat. Med. 2015:394926. doi: 10.1155/2015/394926 26379744 PMC4563062

[B48] Macleod M. R. O'Collins T. Howells D. W. Donnan G. A. (2004). Pooling of animal experimental data reveals influence of study design and publication bias. Stroke 35, 1203–1208. doi: 10.1161/01.STR.0000125719.25853.20 15060322

[B49] McGarry T. Biniecka M. Veale D. J. Fearon U. (2018). Hypoxia, oxidative stress and inflammation. Free Radic. Biol. Med. 125, 15–24. doi: 10.1016/j.freeradbiomed.2018.03.042 29601945

[B50] Michel T. Feron O. (1997). Nitric oxide synthases: which, where, how, and why? J. Clin. Invest. 100, 2146–2152. doi: 10.1172/JCI119750 9410890 PMC508408

[B51] Mitchell S. Vargas J. Hoffmann A. (2016). Signaling via the NFκB system. Wiley Interdiscip. Rev. Syst. Biol. Med. 8, 227–241. doi: 10.1002/wsbm.1331 26990581 PMC8363188

[B52] Mu Q. Yao K. Syeda M. Z. Zhang M. Cheng Q. Zhang Y. . (2023). Ligustrazine nanoparticle hitchhiking on neutrophils for enhanced therapy of cerebral ischemia-reperfusion injury. Adv. Sci. 10:e2301348. doi: 10.1002/advs.202301348 PMC10323616 37078794

[B53] Ni G.-X. Liang C. Wang J. Duan C.-Q. Wang P. Wang Y.-L. (2020). Astragaloside IV improves neurobehavior and promotes hippocampal neurogenesis in MCAO rats though BDNF-TrkB signaling pathway. Biomed. Pharmacother. 130:110353. doi: 10.1016/j.biopha.2020.110353 32682983

[B54] Park J. Y. Pillinger M. H. Abramson S. B. (2006). Prostaglandin E2 synthesis and secretion: the role of PGE2 synthases. Clin. Immunol. 119, 229–240. doi: 10.1016/j.clim.2006.01.016 16540375

[B55] Qi C. Liu Y. Tian Y. Zhang J. Chen X. Zhang P. . (2007b). Effects of tetramethylpyrazine on cell proliferation in dentate gyrus in rat brain after cerebral ischemia-reperfusion injury. J. Xian Jiaotong Univ. Sci. 28, 142–160.

[B56] Qi C. F. Liu Y. Zhang J. S. Tian Y. M. Chen X. L. Zhang P. B. . (2007a). Effect of ligustrazine on nNOS expression and neuranagenesis in adult rats after cerebral ischemia-reperfusion injury. J. South. Med. Univ. 27, 771–774. 17584635

[B57] Ribas V. García-Ruiz C. Fernández-Checa J. C. (2014). Glutathione and mitochondria. Front. Pharmacol. 5:151. doi: 10.3389/fphar.2014.00151 25024695 PMC4079069

[B58] Sadigh-Eteghad S. Geranmayeh M. H. Majdi A. Salehpour F. Mahmoudi J. Farhoudi M. (2018). Intranasal cerebrolysin improves cognitive function and structural synaptic plasticity in photothrombotic mouse model of medial prefrontal cortex ischemia. Neuropeptides 71, 61–69. doi: 10.1016/j.npep.2018.07.002 30054019

[B59] Shao H. He X. Zhang L. Du S. Yi X. Cui X. . (2021). Efficacy of ligustrazine injection as adjunctive therapy in treating acute cerebral infarction: a systematic review and meta-analysis. Front. Pharmacol. 12:761722. doi: 10.3389/fphar.2021.761722 34880757 PMC8646035

[B60] Shi K. Tian D.-C. Li Z.-G. Ducruet A. F. Lawton M. T. Shi F.-D. (2019). Global brain inflammation in stroke. Lancet Neurol. 18, 1058–1066. doi: 10.1016/S1474-4422(19)30078-X 31296369

[B61] Shu M. Dai Y. Song L. Ma D. Liu K. Miao Z. . (2024). Tetramethylpyrazine regulates angiogenesis of endothelial cells in cerebralischemic stroke injury via SIRT1/VEGFA signaling pathway. China J. Chin. Mater. Medica 49, 162–174. doi: 10.19540/j.cnki.cjcmm.20231116.303 38403349

[B62] Siddaway A. P. Wood A. M. Hedges L. V. (2019). How to do a systematic review: a best practice guide for conducting and reporting narrative reviews, meta-analyses, and meta-syntheses. Annu. Rev. Psychol. 70, 747–770. doi: 10.1146/annurev-psych-010418-102803 30089228

[B63] Simon L. S. (1999). Role and regulation of cyclooxygenase-2 during inflammation. Am. J. Med. 106, 37S–42S. doi: 10.1016/S0002-9343(99)00115-1 10390126

[B64] Tabrizi R. Vakili S. Akbari M. Mirhosseini N. Lankarani K. B. Rahimi M. . (2019). The effects of curcumin-containing supplements on biomarkers of inflammation and oxidative stress: a systematic review and meta-analysis of randomized controlled trials: curcumin intake and inflammation. Phytother. Res. 33, 253–262. doi: 10.1002/ptr.6226 30402990

[B65] Tan F. Fu W. Cheng N. Meng D. I. Gu Y. (2015). Ligustrazine reduces blood-brain barrier permeability in a rat model of focal cerebral ischemia and reperfusion. Exp. Ther. Med. 9, 1757–1762. doi: 10.3892/etm.2015.2365 26136889 PMC4471771

[B66] Taylor R. A. Sansing L. H. (2013). Microglial responses after ischemic stroke and intracerebral hemorrhage. Clin. Dev. Immunol. 2013:746068. doi: 10.1155/2013/746068 24223607 PMC3810327

[B67] Teng F. Shen M. Wang L. Gao F. Zhang C. (2021). Ultrasound/microbubble-mediated tetramethylpyrazine for neuroprotection against cerebral ischemia/reperfusion-injured rat brain. Appl. Acoust. 183:108330. doi: 10.1016/j.apacoust.2021.108330

[B68] Tuo Q. Zhang S. Lei P. (2022). Mechanisms of neuronal cell death in ischemic stroke and their therapeutic implications. Med. Res. Rev. 42, 259–305. doi: 10.1002/med.21817 33957000

[B69] Vukic V. Callaghan D. Walker D. Lue L.-F. Liu Q. Y. Couraud P.-O. . (2009). Expression of inflammatory genes induced by beta-amyloid peptides in human brain endothelial cells and in Alzheimer's brain is mediated by the JNK-AP1 signaling pathway. Neurobiol. Dis. 34, 95–106. doi: 10.1016/j.nbd.2008.12.007 19162185 PMC2720310

[B70] Wang Y. Zheng L. Shang W. Yang Z. Li T. Liu F. . (2022). Wnt/beta-catenin signaling confers ferroptosis resistance by targeting GPX4 in gastric cancer. Cell Death Differ. 29, 2190–2202. doi: 10.1038/s41418-022-01008-w 35534546 PMC9613693

[B71] Wang Z. Wu Z. Miao Y. Hao A. Chen H. Zhao S. . (2024). The protective effects of ligustrazine on ischemic stroke: a systematic review and meta-analysis of preclinical evidence and possible mechanisms. Front. Pharmacol. 15:1373663. doi: 10.3389/fphar.2024.1373663 38545549 PMC10965629

[B72] Wells J. M. Gaggar A. Blalock J. E. (2015). Matrix biology. J. Int. Soc. Matrix Biol. 44–46, 122–129. doi: 10.1016/j.matbio.2015.01.016 PMC4838901 25636538

[B73] Xiao X. Liu Y. Qi C. Qiu F. Chen X. Zhang J. . (2010). Neuroprotection and enhanced neurogenesis by tetramethylpyrazine in adult rat brain after focal ischemia. Neurol. Res. 32, 547–555. doi: 10.1179/174313209X414533 20501058

[B74] Yang H. Qi C. Su F. Shan W. Guo A. Wu J. . (2022). Cerebral ischemia/reperfusion injury and pharmacologic preconditioning as a means to reduce stroke-induced inflammation and damage. Neurochem. Res. 47, 3598–3614. doi: 10.1007/s11064-022-03789-5 36327016

[B75] Yang Y. Ren Z. Liang Y. (2009). Study of the expression of cyclooxygenase-2, malonic dialdehyde and the protective effect of tetramethylpyrazine after cerebral ischemic-reperfusion in rat. Acta Anat. Sin. 40, 886–890.

[B76] Ye J. (2015). Beneficial metabolic activities of inflammatory cytokine interleukin 15 in obesity and type 2 diabetes. Front. Med. 9, 139–145. doi: 10.1007/s11684-015-0377-z 25511621 PMC4559335

[B77] Yu B. Ruan M. Zhang Z.-N. Cheng H.-B. Shen X.-C. (2016). Synergic effect of borneol and ligustrazine on the neuroprotection in global cerebral ischemia/reperfusion injury: a region-specificity study. Evid. Based Complement. Alternat. Med. 2016:4072809. doi: 10.1155/2016/4072809 27547227 PMC4983362

[B78] Yu T. Guo X. Zhang Z. Liu R. Zou L. Fu J. . (2016). Meta-analysis of the clinical effectiveness and safety of ligustrazine in cerebral infarction. Evid. Based Complement. Alternat. Med. 2016:3595946. doi: 10.1155/2016/3595946 27738442 PMC5050365

[B79] Zhang Q. Qian Z. Pan L. Li H. Zhu H. (2012). Hypoxia-inducible factor 1 mediates the anti-apoptosis of berberine in neurons during hypoxia/ischemia. Acta Physiol. Hung. 99, 311–323. doi: 10.1556/APhysiol.99.2012.3.8 22982719

[B80] Zhu X.-L. Xiong L.-Z. Wang Q. Liu Z.-G. Ma X. Zhu Z.-H. . (2009). Therapeutic time window and mechanism of tetramethylpyrazine on transient focal cerebral ischemia/reperfusion injury in rats. Neurosci. Lett. 449, 24–27. doi: 10.1016/j.neulet.2008.09.007 18790005

