# Supplementary material for: Exploring the neuroprotective potential of ligustrazine: a preclinical meta-analysis and machine learning perspective on cerebral ischemia-reperfusion injury
Source: Front Neurosci. 2026 May 11;20:1754510. doi: 10.3389/fnins.2026.1754510 (PMC13199256; doi:10.3389/fnins.2026.1754510)
Supplement: Supplementary file 1 [file Data_Sheet_1.docx]

**Supplementary Material**

**Exploring the neuroprotective potential of ligustrazine: A preclinical meta-analysis and machine learning perspective on cerebral ischemia-reperfusion injury**

Lang Tu^a,†^, Yimiao Luo^b,†^, Xinyi Xu^c^, Huiling Xiong^d^, Zilong Zhang^a^, Xiaoyuan Zhou^a,*^

^a^ West China school of public health/ West China Fourth Hospital, Sichuan University, Chengdu, 610041, China

^b^ School of Chinese Medicine, The University of Hong Kong, Hong Kong Special Administrative Region, China.

^c^ School of Public Health, Chengdu University of Traditional Chinese Medicine, Chengdu, 610075, China

^d^ Acupuncture and Tuina School, Chengdu University of Traditional Chinese Medicine, Chengdu, 610075, China

^†^ Co-first authors

^*^ Corresponding author: Xiaoyuan Zhou, West China school of public health/ West China Fourth Hospital, Sichuan University, Chengdu, 610041, China, Email address: [zhouxyuan@scu.edu.cn](mailto:zhouxyuan@scu.edu.cn).

Supplementary Table 1 Retrieval strategies

| **Database** | **Number** | **Search terms** |
| --- | --- | --- |
| **PubMed** | #1 | **"ligustrazine"OR"tetramethylpyrazine"OR"tetramethylpyrazine hydrochloride"OR"Liqustrazine"OR"TMPZ"OR"tetramethylpyrazine nitrone"OR "chuanxiongzine"OR"3-hydro-2,2,5,6-tetramethylpyrazine"OR"tetramethylpyrazine diphenylmethylpiperazidine"OR"3,5,6-trimethylpyrazinecarboxylic acid"OR"C8H12N2"OR"2,3,5,6-Tetramethylpyrazine"OR"2,3,5,6-Tetramethyl"** |
|  | #2 | **"Cerebral Infarction"OR"Acute Ischemic Stroke"OR"Cerebral Infarct"OR"Infarction, Cerebral"OR"Infarctions, Cerebral"OR"Cerebral Infarct" "Cerebral Infarcts"OR"Ischemic Stroke"OR "MCAO" OR "OGD/R"OR"cerebral ischemia reperfusion injury"** |
|  | #3 | #1 AND #2 |
| **EMBASE** | #1 | **‘ligustrazine’:ti,ab,kw OR ‘tetramethylpyrazine’:ti,ab,kw OR 'tetramethylpyrazine hydrochloride':ti,ab,kw OR 'liqustrazine':ti,ab,kw OR 'tmpz':ti,ab,kw OR 'tetramethylpyrazine nitrone':ti,ab,kw OR 'chuanxiongzine':ti,ab,kw OR '3-hydro-2,2,5,6 tetramethylpyrazine':ti,ab,kw OR 'tetramethylpyrazine diphenylmethyl piperazidine':ti,ab,kw OR '3,5,6-trimethylpyrazinecarboxylicacid':ti,ab,kw OR 'c8h12n2':ti,ab,kw OR '2,3,5,6-tetramethylpyrazine':ti,ab,kw OR '2,3,5,6 tetramethyl':ti,ab,kw** |
|  | #2 | **‘cerebral infarction’:ti,ab,kw OR ‘acute ischemic stroke’:ti,ab,kw OR ‘infarction, cerebral’:ti,ab,kw OR ‘infarctions, cerebral’:ti,ab,kw OR ‘cerebral infarct’:ti,ab,kw OR ‘cerebral infarcts’:ti,ab,kw OR ‘ischemic stroke’:ti,ab,kw OR ‘mcao’:ti,ab,kw OR ‘Oxygen glucose deprivation’:ti,ab,kw OR ‘re-oxygenation’:ti,ab,kw OR ‘cerebral ischemia reperfusion injury’:ti,ab,kw** |
|  | #3 | #1 AND #2 |
| **Web of Science** | #1 | **(TS=((Ligustrazine) OR (tetramethylpyrazine) OR ("tetramethylpyrazine hydrochloride") OR (Liqustrazine) OR (TMPZ) OR ("tetramethylpyrazine nitrone") OR (chuanxiongzine) OR ("3-hydro-2,2,5,6-tetramethylpyrazine")OR ("tetramethylpyrazine diphenylmethyl piperazidine")OR ("3,5,6-trimethylpyrazinecarboxylic acid") OR (C8H12N2)OR ("2,3,5,6-Tetramethylpyrazine") OR ("2,3,5,6-Tetramethyl"))) AND TS=(("Cerebral Infarction") OR ("Acute Ischemic Stroke") OR ("Cerebral Infarct") OR ("Infarction, Cerebral") OR ("Infarctions, Cerebral") OR ("Cerebral Infarct") OR ("Cerebral Infarcts") OR ("Ischemic Stroke") OR(MCAO) OR (OGD/R) OR ("cerebral ischemia reperfusion injury"))** |
| **Cochrane Library** | #1 | **MeSH descriptor: [Cerebral Infarction] this term only** |
|  | #2 | **(Cerebral Infarction OR Acute Ischemic Stroke OR Cerebral Infarct OR Infarction, Cerebral OR Infarctions, Cerebral OR Cerebral Infarct OR Cerebral Infarcts OR Ischemic Stroke OR MCAO OR OGD OR cerebral ischemia reperfusion injury):ti,ab,kw** |
|  | #3 | #1 OR #2 |
|  | #4 | **MeSH descriptor: [tetramethylpyrazine] this term only** |
|  | #5 | **(Ligustrazine OR tetramethylpyrazine OR tetramethylpyrazine hydrochloride OR Liqustrazine OR TMPZ OR tetramethylpyrazine nitrone OR chuanxiongzine OR 3 hydro 2,2,5,6 tetramethylpyrazine OR tetramethylpyrazine diphenylmethyl piperazidine OR 3,5,6 trimethylpyrazinecarboxylic acid OR C8H12N2 OR 2,3,5,6 Tetramethylpyrazine OR 2,3,5,6 Tetramethyl):ti,ab,kw** |
|  | #6 | #4 OR #5 |
|  | #7 | #3 AND #6 |

Supplementary Table 2 List of molecular and cellular mechanisms driving the protective effect of Ligustrazine against cerebral I/R injury in all 21 studies

| Author | Year | ***Proposed mechanisms*** |
| --- | --- | --- |
| Liao.et al | 2004 | Ligustrazine could effectively treat ischemia-reperfusion injury by inhibiting glial cell production of COX-2 and PGE2, reducing leukocyte migration and production of pro-inflammatory factors, and inhibiting immune activation and anti-inflammatory effects. |
| Hsiao.et al | 2006 | In the ischemic brain, nitrotyrosine and inducible nitric oxide synthase (iNOS) were significantly inhibited to avoid the toxicity of excessive NO to brain tissue, and ligusstrazine was also effective in scavenging reactive oxygen species. |
| Chang.et al | 2007 | Ligustrazine exerted anti-inflammatory and anti-apoptotic effects by inhibiting the activation of HIF-1α and TNF-α and then down-regulating the expression of caspase-3. |
| Qi.et al (a) | 2007 | Ligustrazine alleviated delayed neuronal injury and neuronal edema by antagonizing excitatory amino acid toxicity and altering NO levels in the late reperfusion period, and promoted the proliferation of dentate neural stem cells in the hippocampus. |
| Qi.et al (b) | 2007 | Ligustrazine could inhibit the expression of nNOS in the early stage after ischemic brain injury, affect the glutamate metabolism in the excitatory amino acid NMDA receptor pathway, which promoted the proliferation of SVZ and DG nerve cells. |
| Jia.et al | 2008 | Ligustrazine upregulated TrX expression through the activation of thioredoxin system, thereby scavenging free radicals and enhancing antioxidant activity. |
| Yang.et al | 2009 | Ligustrazine could down-regulate the expression of COX-2 and reduce the content of MDA to treat cerebral I/R injury. |
| Zhu.et al | 2009 | Ligustrazine could down-regulate the expression of nNOS and iNOS and stimulate cell proliferation and differentiation in SVZ and striatum. |
| Xiao.et al | 2010 | Ligustrazine inhibits the release of endogenous amino acids, including aspartic acid, glutamic acid, taurine acid, and γ-amino butyric acid, following I/R injury. |
| Han.et al | 2014 | Ligustrazine upregulated MAP-2 level in the peri-infarct area, improved the dendritic plasticity, and finally promoted the recovery of neurological function. |
| Lin.et al | 2015 | Ligustrazine could reduce the permeability of the blood-brain barrier after I/R, prevent damage to the tight junction proteins occludin and claudin-5 in the brain barrier, and it down-regulated the expression and activity of matrix metalloproteinases responsible for extracellular matrix and tight junction degradation in the rat brain. |
| Tan.et al | 2015 | The protective effect was achieved by protecting the integrity of the blood-brain barrier, containing reduced expression of MMPs and damage to tight junction proteins. |
| Yu.et al | 2016 | The therapeutic effect was achieved by improving microcirculation, inhibiting inflammatory response, anti-oxidative stress, and anti-apoptosis. |
| Liao.et al | 2018 | NM |
| Ding.et al | 2019 | Ligustrazine activates the eNOS/NO signaling pathway and the PI3K/Akt pathway to stimulate the increase of NO and achieve the therapeutic effect of ischemia-reperfusion injury. |
| Cao.et al | 2020 | Ligustrazine promoted neurogenesis and inhibited inflammation by up-regulating the expressions of α-tubulin and nestin and down-regulating the expressions of GFAP and IL-1 |
| Jin.et al | 2021 | Ligustrazine could downregulate the levels of TNF-α, IL-1β, IL-6 and MMP-9 and upregulate the expression of MCPIP1, thereby inhibiting inflammation, anti-apoptosis and protecting the blood-brain barrier. |
| Lin.et al | 2021 | Ligustrazine could up-regulate SYP expression and improve prominent ultrastructure remodeling |
| Teng.et al | 2021 | NM |
| Li.et al | 2022 | Ligustrazine inhibited the increase of circ_0008146 and upregulated the expression of miR-709, which further downregulated the expression of Cx3cr1, reduced the contents of IL-6 and TNF-α, and reduced nerve cell death |
| Mu.et al | 2023 | NM |
| Shu.et al | 2024 | Ligustrazine regulated angiogenesis of endothelial cells via SIRTl/VEGFA signaling pathway. |
| Chen.et al | 2024 | Ligustrazine could ameliorate abnormal mitochondrial dynamics by reducing Drp1 SUMO-1ylation and increasing Drp1 SUMO-2/3ylation. |


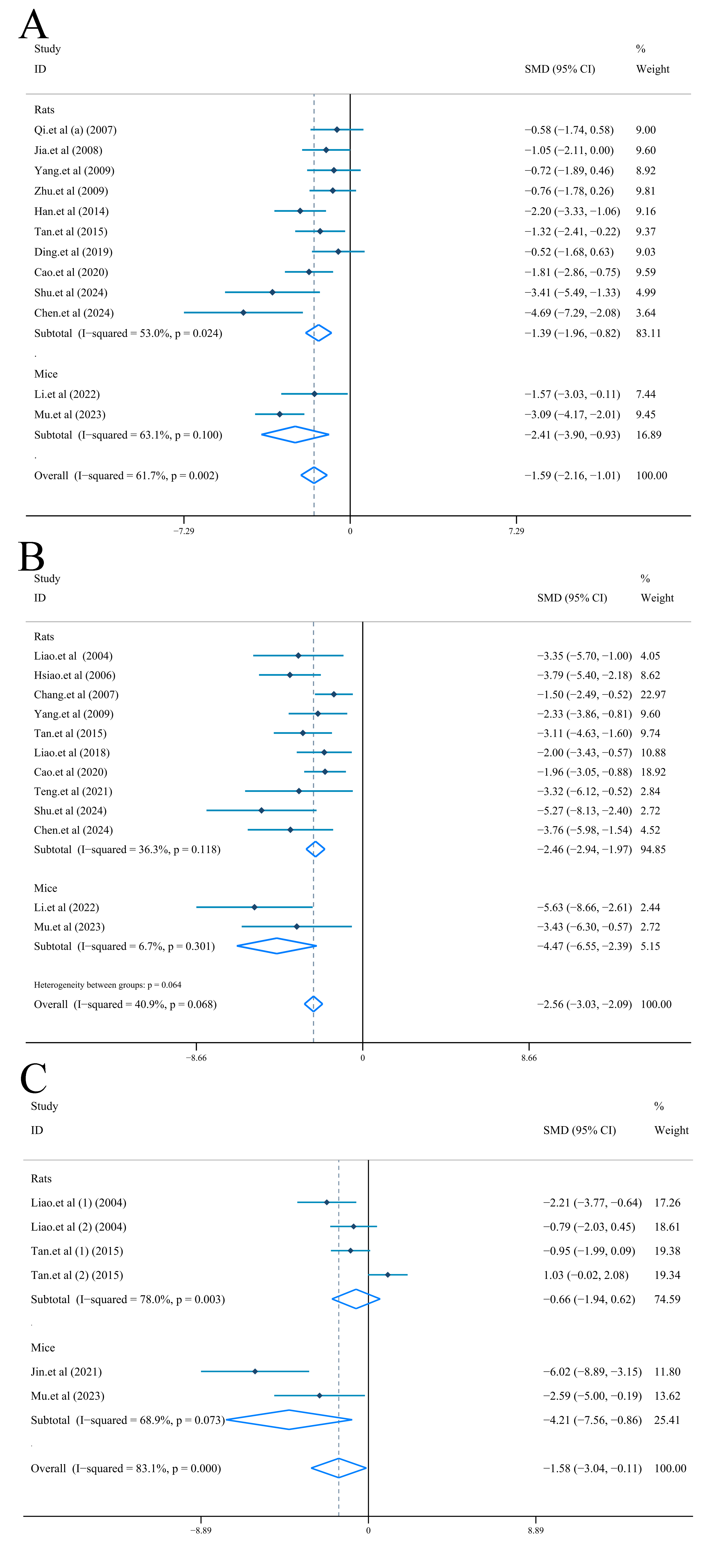


Supplementary Figure 1 Subgroup analysis of the indicators based on the rodent species. (A) neurological function scores based on Longa criteria; (B) Infarct volume (%); (C) Brain content water.





Supplementary Figure 2 Publication bias and sensitivity analysis of neurological function scores based on Longa criteria. (A)Publication bias; (B) Sensitivity analysis.





Supplementary Figure 3 Publication bias and sensitivity analysis of Infarct volume (%). (A)Publication bias; (B) Sensitivity analysis.





Supplementary Figure 4 Publication bias and sensitivity analysis of Infarct volume. (A)Publication bias; (B) Sensitivity analysis.





Supplementary Figure 5 Publication bias and sensitivity analysis of brain content water. (A)Publication bias; (B) Sensitivity analysis.


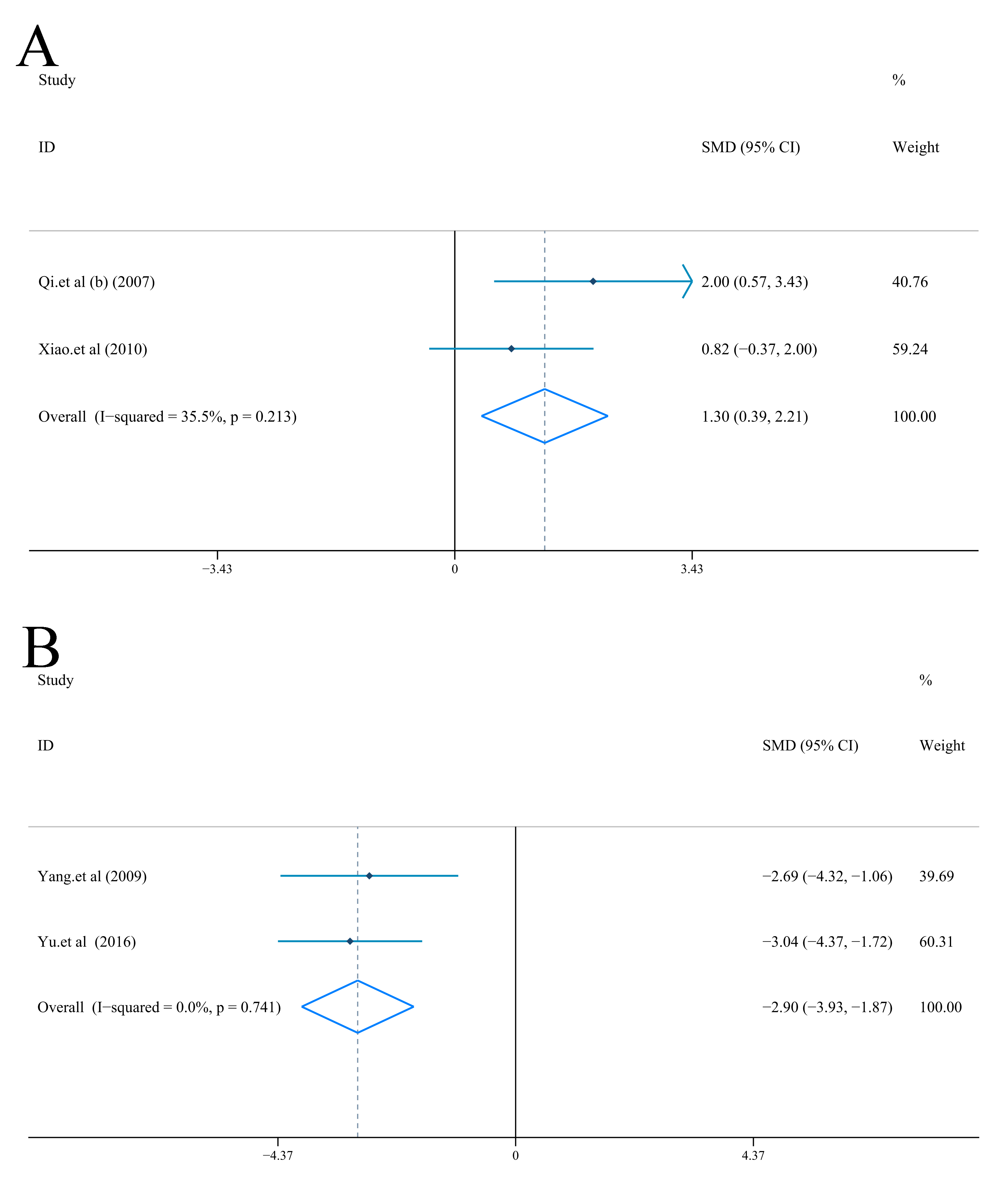


Supplementary Figure 6 Forest plot (effect size and 95% CI) summarizing the effect of Ligustrazine on the indicators. (A) Cells in the subventricular zone; (B) Content of MDA in cerebral cortex.


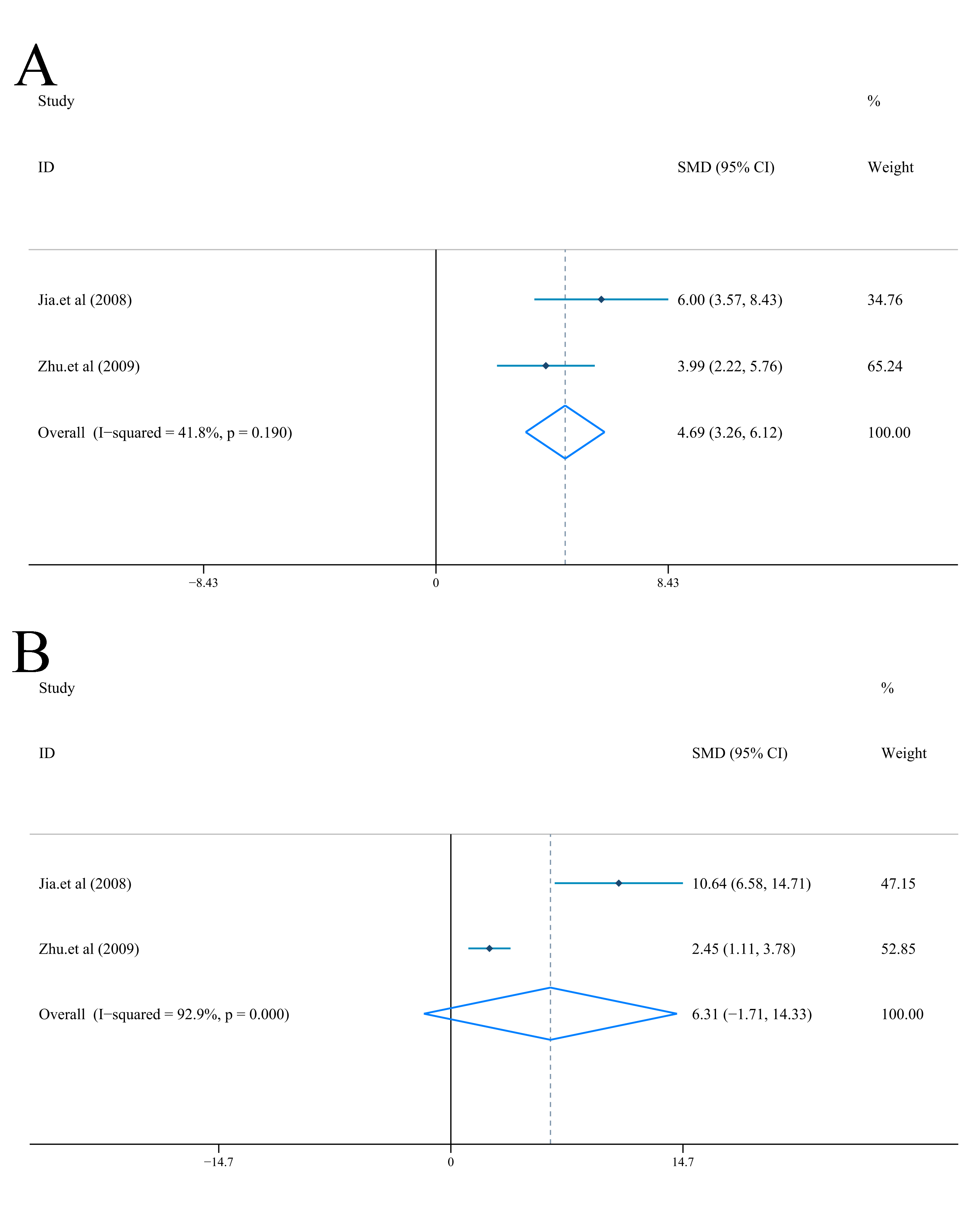


Supplementary Figure 7 Forest plot (effect size and 95% CI) summarizing the effect of Ligustrazine on the thioredoxin. (A) Thioredoxin-1; (B) Thioredoxin-2.


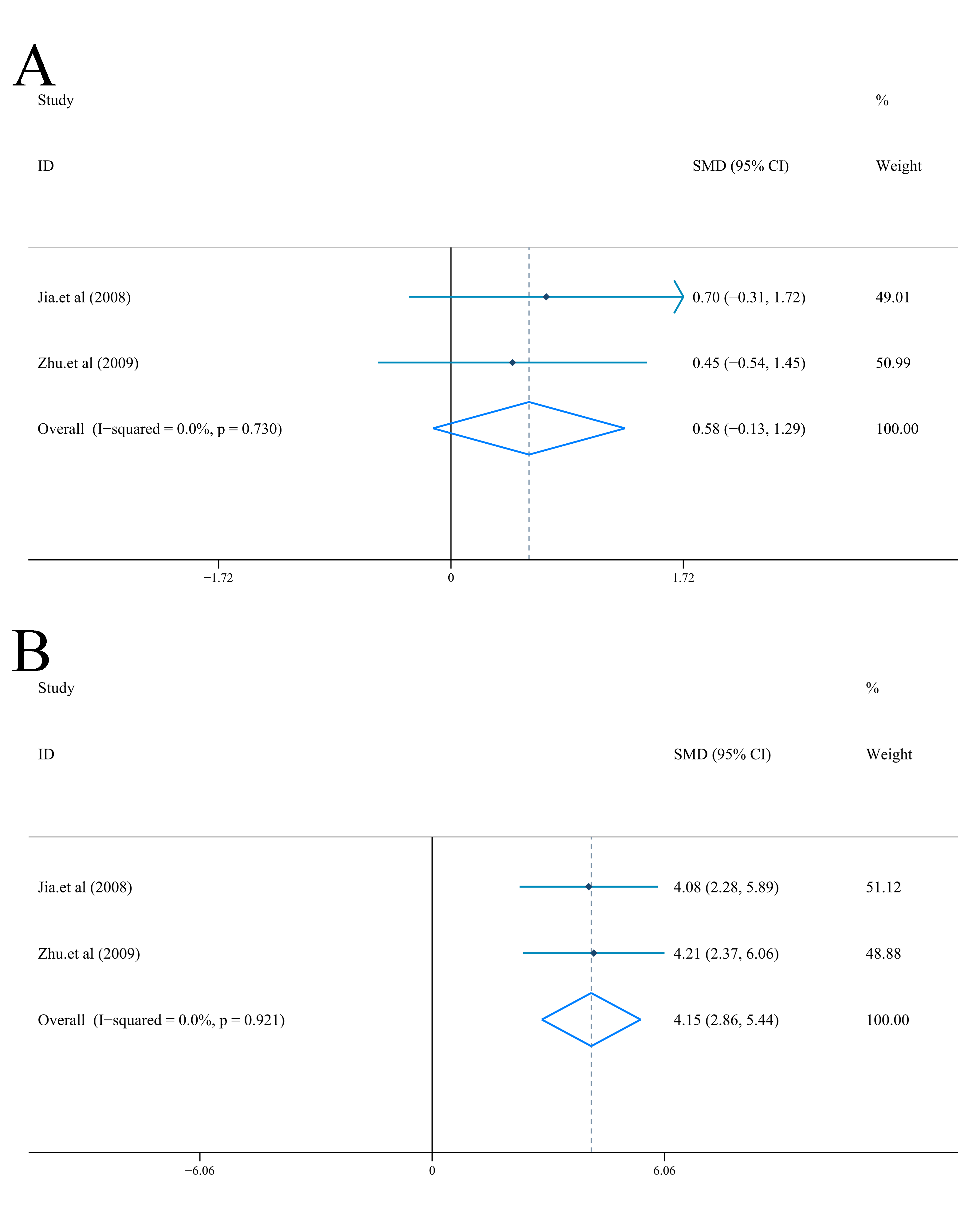


Supplementary Figure 8 Forest plot (effect size and 95% CI) summarizing the effect of Ligustrazine on the thioredoxin reductase. (A) Thioredoxin reductase 1; (B) Thioredoxin reductase 2.
